# Microbiological information in the precipitate and supernatant fractions after wastewater centrifugation—loss or profit for hygiene, epidemiology, and environmental surveillance?

**DOI:** 10.3389/fpubh.2026.1897003

**Published:** 2026-10-26

**Authors:** Karol Korzekwa, Oliwia Obuch-Woszczatyńska, Klaudia Bylińska, Agnieszka Kauc, Małgorzata Krzyżowska

**Affiliations:** 1 Department of Medical and Environmental Microbiology, Military Institute of Hygiene and Epidemiology, Warsaw, Poland; 2 Laboratory of Hygiene and Environmental Sanitary Surveillance, Military Institute of Hygiene and Epidemiology, Warsaw, Poland; 3 Parasitology Laboratory, Military Institute of Hygiene and Epidemiology, Warsaw, Poland; 4 Division of Pharmacology and Toxicology, Department of Preclinical Sciences, Institute of Veterinary Medicine, Warsaw University of Life Sciences, Warsaw, Poland

**Keywords:** bio-surveillance, drug-resistance, ESKAPE, re-emerging pathogens, SARS-CoV-2, wastewater-based epidemiology

## Abstract

**Objectives:**

Wastewater-based bio-surveillance is essential for monitoring pathogens in communities. This study examines whether supernatant and the sediment left after centrifuging sewage should be tested separately or together. The goal is to assess how useful sediment information is for making accurate public health decisions.

**Materials and methods:**

Total nucleic acids were isolated from wastewater inflows and outflows in Warsaw, including contributions from healthcare facilities, the airport, and residential areas, to detect SARS-CoV-2, ESKAPE pathogens, re-emerging pathogens, alarming pathogens, drug-resistance patterns, and pathotypes during two consecutive waves of COVID-19. Total DNA was extracted from both sewage water and solid phases using vacuum pressure and semi-automated methods. This DNA was then amplified using RT-qPCR and qPCR in *in vitro* diagnostic (IVD) tests. The analysis focused on the presence of E, N1, and N2 markers for SARS-CoV-2, sequences indicating pathotypes (such as *stx*, *eae*, *ipaH*, *elt*, and *est*), and genome determinants of antibiotic resistance genes in bacteria (including *van*, *bla*, *mec*, SA442/CoA, SCCmec-orfX, and *mcr*).

**Results:**

A two-year study involving 80 locations in an urban catchment revealed both qualitative and quantitative differences in the phases following centrifugation of nearly 1,000 sewage samples. In 350 grams of sediment and over 60 micrograms of various TNA fractions, we observed frequent positive results in the sediments, while these results were absent in the supernatant (19:7).

**Conclusion:**

The careful consideration of post-centrifugation phases simplifies probe selection methods, enhances the accuracy of epidemiological inferences, and enables effective responses to public health challenges, whether during normal operations or times of crisis. Additionally, quickly and accurately determining the approximate number of infected individuals can help prevent the manipulation of epidemiological data.

## 1 Introduction

The isolation of total nucleic acids (TNA) for wastewater-based epidemiology (WBE) has become standard practice. Various methods are employed to monitor wastewater, with quantitative polymerase chain reaction (qPCR) being one of the primary techniques (1). This method offers significant advantages, including high sensitivity, specificity, reproducibility, cost-effectiveness for large-scale screening, automation, and high throughput. These benefits are also essential for diagnostic applications. Recent optimizations in qPCR have led to the development of rapid, multiplex *in vitro* diagnostic (IVD) tests. These tests are increasingly recognized for their use in clinical settings and for monitoring public health during epidemics. They play a vital role in sanitary and pre-epidemiological surveillance by enabling the rapid identification of microorganisms in the environment. The process involves the extraction and purification of DNA, RNA, and proteins using a single, fast kit, which supports preventive measures. Despite some authors believing that traditional step-by-step methods are more effective than kits, it’s important to acknowledge that kits are increasingly preferred in laboratory settings. Their ease of use and efficiency make them a valuable choice for researchers. When combined with new sequencing techniques and biosensor support, simple IVD kits emerge as convenient tools for rapid screening in health and environmental protection (2). However, challenges remain when working with sewage matrices, leading to poor repeatability and uncertainty in multiplex reaction results. The presence of strong interfering and disturbing compounds in the sewage background during real-time reactions, along with potential errors in the initial centrifugation stage, poses significant problems (3).

The impact of various components on the quality of wastewater analysis relies heavily on the methods used for homogenization, clarification, and concentration. When significant amounts of TNA are anticipated, testing as little as 1 mL of raw wastewater may be sufficient. To continuously monitor bioindicators present near or below the method’s limit of quantification, bulk samples (500 mL to 5 L) are typically concentrated and purified down to approximately 1 mL. The techniques for concentration and purification can vary and include methods such as centrifugation, ultrafiltration (UF), bag-mediated filtration systems (BMFS), the electronegative membrane vortex (EMV) procedure, skim milk flocculation, and polyethylene glycol precipitation (PEG) (4, 5). Wastewater that is rich in bioindicators and solid substances often undergoes thermal or physical pre-treatment, such as sonication, before concentration and clarification processes are applied.

The thermal conditions, time, and physics of the centrifugation process remain crucial. The upper phases obtained after centrifugation in classical methods serve for further purification of DNA/RNA, the interphases (proteins) and the lower phases provide compounds that are soluble in halogenoalkanes and phenols. The first stage of many procedures for nucleic acid extraction from wastewater involves centrifugation under non-stringent conditions, allowing the obtaining of upper and lower phases, both of which are valuable (6). However, many investigations prefer the upper (water) phase rather than the lower one (pellet). In many cases, accurate selection of molecular markers determines the result (7, 8).

In this paper, the methodological significance of the two phases, percentage and importance, was verified in accordance with five hypotheses. Method verification was performed across centrifugation, vacuum extraction, mini-column clean-up, and semi-automated extraction of wastewater samples. Investigations were conducted during SARS-CoV-2, *Enterococcus faecium*, *Staphylococcus aureus*, *Klebsiella pneumoniae*, *Acinetobacter baumannii*, *Pseudomonas aeruginosa*, and *Enterobacter* species (shortly ESKAPE) tracing, re-emerging pathogens surveillance, and antimicrobial chemotherapeutics tracing in a municipal sewage catchment. The abundance of literature on SARS-CoV-2 analyses is accompanied by little data on bacteria and molecular features about resistance to chemotherapeutics in wastewater during the epidemiological crisis in large urban locations. Testing and optimizing IVD kits for wastewater analysis, as well as identifying valuable bioindicators that meet the requirements of Directive (EU) 2024/3019 from the European Parliament and Council, is a patchwork approach that addresses existing gaps and fulfils real needs.

To address the question in the title, this study leveraged precipitate and supernatant obtained through centrifugation to highlight methodological factors essential for accurately estimating the number of infected individuals or pre-epidemic sources.

## 2 Materials and methods

### 2.1 Sample collection

The sewage samples were collected from four types of catchment areas: transportation hubs, healthcare facilities, housing estates, and inflows and outflows from wastewater treatment plants. This covered an area of 517 square kilometers and served nearly 2 million equivalent residents. The outflows from the wastewater treatment plants represented treated sewage following the biological processing. Sampling at these collection points was conducted using composite sampling methods. Time-proportional and flow-proportional methods were applied to collect a 500 mL representative sample from autosamplers (WaterSam 312, 4,121,541 and 4,121,352, Germany; Buhler 4,011, type BU4011.60.11111, HACH LANGE, UK). Sampling was conducted from April 2023 to December 2024 during standard COVID-19 surveillance in Warsaw, Poland. In total, 862 samples were chosen from 20-month-long analyses. During the specified period, samples were tested weekly for the presence of SARS-CoV-2 (see Supplementary Table 1 for dates in the first column). Additionally, bacteriological testing, which included analysis for drug-resistance genes, was conducted on a monthly basis (refer to Supplementary Table 4 for dates in the first column).

Samples from the catchment area were collected from inspection wells between 7:00 a.m. and 4:00 p.m. Sampling at wastewater treatment plants (WWTPs) took place on Monday at 6:30 a.m. and Tuesday at 7:00 a.m. Raw sewage samples were collected proportionally based on both time and flow, with 150 mL samples taken every 30 min. In contrast, treated sewage samples were collected based on volume, specifically between 100 and 150 mL for every 100 to 1,000 square meters of sewage, depending on the size of the treatment plant. Both raw and treated sewage were collected in SCHOTT-type glass bottles with screw-on caps and were transported and stored at 4 °C (± 2) for no longer than 14 h until extraction.

### 2.2 Total nucleic acid (TNA) isolation, extraction, and purification

A 40-mL wastewater sample was separated into supernatant and pellet by centrifugation (3,000 × g, 10 min, room temperature). The supernatant was transferred to a clean 50 mL Falcon-type tube, the pellet was dried for 1 h at room temperature, then weighed, and processed separately, according to a slightly modified Wizard Enviro Total Nucleic Acid Kit procedure (Promega – A2991, Madison, WI, USA). However, the pellets obtained after the centrifugation of samples, representing treated wastewater discharged into river receivers, were discarded and did not undergo further analysis due to the negligible amount and sporadic presence of the precipitates in outflows.

After isolation from 40 mL of raw sewage and 5 mL of pellet diluted in nuclease-free water, the final sample was 1 mL. After the second stage of extraction, with cleaning, the 1 mL sample was reduced to 25 μL and stored at −20 °C (± 2) no longer than 14 h, until further processing. Simultaneously, for selected samples, extraction, concentration, and purification of the whole material using the Maxwell RSC instrument (Promega – AS4500, Madison, WI, USA) with Pathogen Total Nucleic Acid Kit and Faecal Microbiome DNA Kit (Promega – AS1890; AS1700, Madison, WI, USA), were performed. The quantity and the quality of the TNA were estimated spectrophotometrically (Nabi UV/Vis Nano Spectrophotometer NB1-A-180404; MicroDigital Co., Korea). Samples underwent thawing for 15 min, and measurements were made in a volume of 1 μL for 260/280 and 260/230 absorbance ratios.

During the analysis, minor methodological changes were introduced in the first phase of TNA isolation. Specifically, the ethanol concentration was adjusted from 95 to 96%, and the centrifugation temperature was modified from room temperature (ranging from 20 °C to 26 °C) to 18 °C. These changes were not part of the original design assumptions but were implemented for technical reasons.

### 2.3 Amplification procedures

After the isolation and treatment of TNA, wastewater samples were amplified to detect the presence of the SARS-CoV-2 virus, as well as bacteria that indicate the sanitary and hygienic status of the capital’s catchment area. We utilized dedicated kits specifically designed for SARS-CoV-2, which included pre-formulated reagents, standards, and calibrations. Additionally, IVD sets for detecting bacteria and antimicrobial agents were also employed.

#### 2.3.1 Viruses

Quantitative reverse transcription polymerase chain reaction (RT-qPCR) was used for material analysis. The reactions were performed according to the GoTaq Enviro Wastewater SARS-CoV-2 Kits (Promega – AM2100, AM2110, AM2120, AM2130, Madison, WI, USA). Amplification was performed in a 10 μL reaction mixture according to a standard thermal profile, i.e., reverse transcription (at 45 °C for 15 min), transcription inactivation (at 95 °C for 2 min), denaturation (at 95 °C for 15 min), and probe/primer annealing with product extension (at 62 °C for 1 min). Previously selected E, N1, and N2 genes for SARS-CoV-2, using the FAM fluorophore, were used in the reaction mixtures. Selected reactions were performed as a control in the presence of the E gene in the reaction mixture. The presence and copy number of the 68-bp RNA region of the pepper-spotted mosaic virus (PMMoV) in the wastewater (Cy5 fluorophore) was an internal control. The presence and copy number of the 285-bp sequence (VIC fluorophore) were used as an internal amplification control. A dilution series for SARS-CoV-2 (N + E) and PMMoV RNA served as the analytical standard. Amplification of PCR samples by QuantStudio 5 Real-Time system (Thermo Fisher Scientific, Waltham, MA, USA) on optical 96-well reaction plates (Applied Biosystems – MicroAmp with barcode, 10,407,314, Thermo Fisher Scientific, Waltham, MA, USA) was performed. Comparative amplification included using the CFX96 Touch Deep Well Real-Time PCR (Bio-Rad Laboratories, Hercules, CA, USA) to control the amplification process for selected samples.

#### 2.3.2 Bacteria

Following TNA isolation and quantification, samples (20–50 ng/μL each) were amplified individually and pooled for downstream analysis. TNA was amplified in duplicate qPCR reactions, followed by IVD diagnostic tests (Vitassay Healthcare S. L. U., Huesca, Spain; Viasure V-smart (VS), multiplex real-time PCR tests, Certest Biotec S. L, Spain). In the amplification reactions, markers revealing enterobacterial pathotypes (*stx*, *eae*, *ipaH*, *elt*, and *est*), drug resistance of cocci and enterobacteria (*van*, *bla*, *mec*, SA442/CoA, SCCmec-orfX, and *mcr*), and species-specific markers (*inv*, *yst*, and 16SrDNA) were used (ref numbers from 7,041,010 to7041071; ref. num. VS-VAN112L, VS-CPE112L, VS-MSA112L). Ambiguous results underwent confirmation in a third reaction. Positive Ct for sample reactions were ≤40 (internal controls 35 < Ct < 37). If there was no signal in the NTC control and the internal control (IC) was functioning correctly, a CT result <40 for the tested sample was considered specific for that sample, confirming the absence of cross-contamination. At high pathogen load levels, ‘qPCR Colour Compensation’ allowed for the correction of bleed-over (fluorescence bleed-over) in multiplex assays, preventing false-positive results (digital contamination). Amplification with the QuantStudio5 Real-Time system (Thermo Fisher Scientific, Waltham, MA, USA) was performed. No confirmation by subsequent steps using other amplification procedures was planned.

### 2.4 Hypothesis for testing, methodology of inference, and statistics

The analyses were categorized into the following sections: (i) pellet weight; (ii) the quality of TNA following centrifugation; (iii) the percentage of viral genome copies in both the pellet and supernatant, with a focus on SARS-CoV-2 analysis; (iv) the frequency of positive reactions to the presence of a marker for specific bacterial groups in the pellet and supernatant; and (v) the similarities and differences in the methods of isolation, extraction, and purification of nucleic acids used in the study. Based on these stages, five research hypotheses were established. H1: There are significant differences in the weight of pellets across different time periods and collection points during the investigation. H2: The weight of the pellet is proportional to the concentration of TNA obtained after extraction, isolation, and purification. H3: The quantity and quality of TNA isolated from the pellet differs from that of the supernatant in each sample. H4: The frequency of positive results in the amplification reaction differs between the pellet and the supernatant. H5: Omitting the analysis of post-centrifugation precipitates in pre-epidemiological surveillance affects the results of the surveillance and potentially their interpretation.

The catchment area was divided into the transportation hubs (AH), health sector utilities (HCU), housing estates (HE), and inflows to wastewater treatment plants (WTP). Viral markers, such as c/μL (copies per microliter) for E, and N genetic markers were combined (in the pellet and the supernatant) before and after the normalization process according to the Promega technical manual (TM661). The presence of marker genes revealed in IVD qPCR reactions indicated the presence of a given group of bacteria (or antimicrobial traits) at sampling points, which were then encoded as the 0–1 matrix.

Statistical processing was supported by Jamovi 2.6.44.0, StatPlus LE 7.7.0 (AnalystSoft Inc. version 7), and Statistica 5.0 (StatSoft Inc.) with the assistance of tips by Duthie (9). For further statistical analysis, normality tests, descriptive statistics, Pearson, Spearman correlations, and ANOVA (several one-way tests) were conducted to describe the results (Statistica 5.0; StatPlus LE 7.7.0).

## 3 Results

### 3.1 The yield of the pellet and basic characteristics

The total amount of sediments tested after centrifugation was nearly 350 g, and the average sample yielded 0.46 g (SE 0.08). The analysis revealed that the lowest pellet weight was 0.01 g, which was obtained from samples collected in July 2023. In contrast, the highest sediment weight was 2.48 g, sourced from samples collected in April 2024 (see Figure 1; Supplementary Tables 1A,B). Pellet weights generally did not differ from each other within a given site and across months, with some exceptions (Supplementary Figure 1, Supplementary Table 2). Pearson correlation analysis revealed multiple significant relationships based on one-tailed tests, including 11 strong positive (*p* < 0.001) and 6 negative correlations (*p* < 0.01). Sediment weights in the months with the highest correlation were identical and were mostly positively correlated with each other in June 2023 and October 2024 and in July and November 2024 (Supplementary Table 3). In turn, the increase in sediment weight in July 2023 was accompanied by a decrease in weight in August 2023 (r = −0.80; *p* < 0.01). Typically, sediments and pellets after centrifugation had a classic color, ranging from light beige to brown. However, there were also sediments and sewage with a color ranging from milky white, dark lemon, to dark orange with a characteristic ‘pharmaceutical’ or ‘anise’ smell rather than fecal (Supplementary Figure 2).

**Figure 1:**
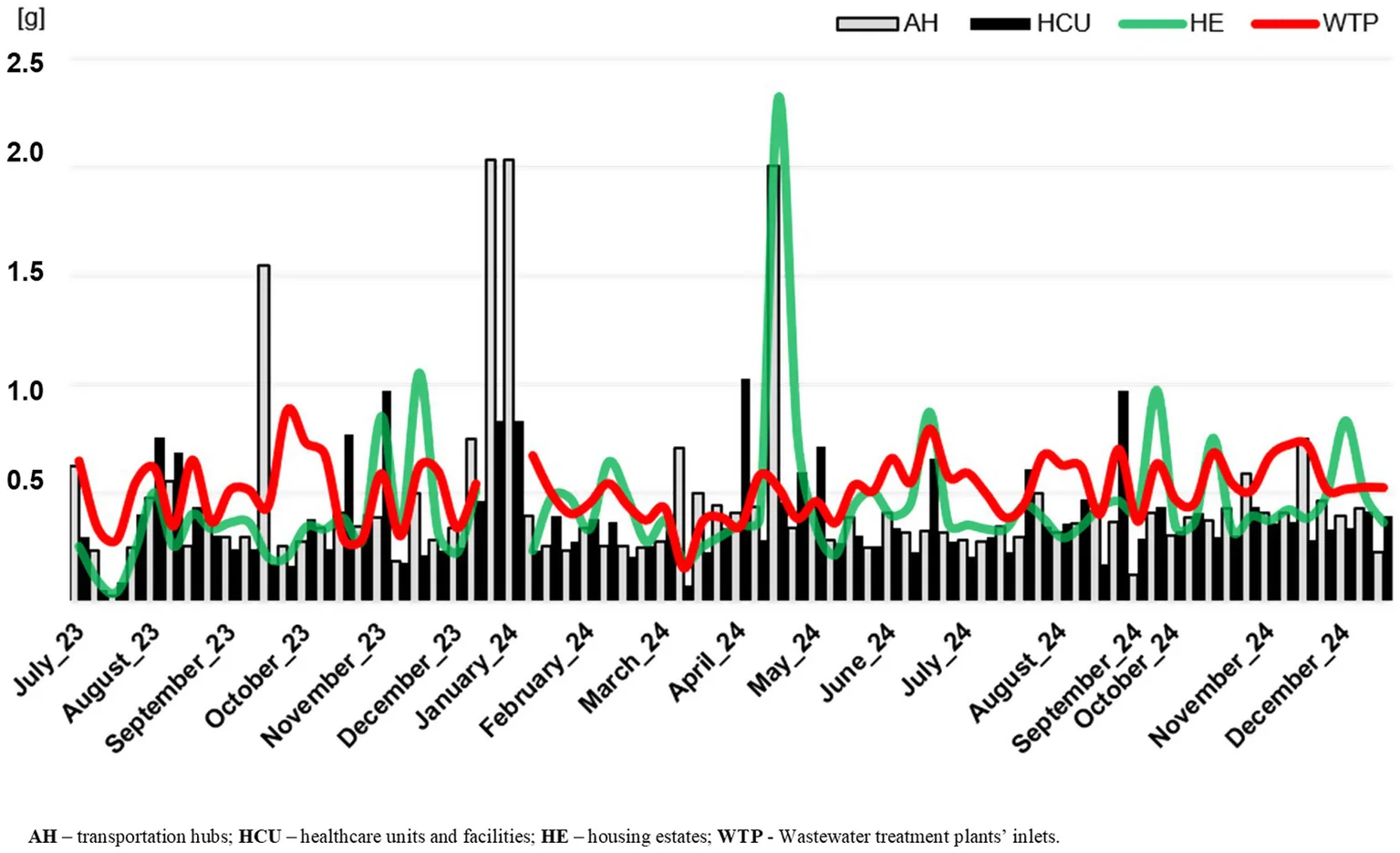
Pellet weights after centrifugation, divided by month, for the main catchment areas and critical infrastructure. AH, transportation hubs; HCU, healthcare units and facilities; HE, housing estates; WTP, Wastewater treatment plants’ inlets.

### 3.2 TNA quantity and quality in fractioned samples

The differences (quantitative and qualitative) in the isolated TNA were disclosed in the aqueous (supernatant) and precipitant (pellet) fractions. In total, nearly 64 μg of TNA (av. 229 ng/μL) was isolated with an average quality of 2.25 (A260/280) and 1.70 (A260/230) (Table 1A). The total amount of TNA isolated from the pellets (51 ng/μl) was almost 8 times lower than that obtained from the supernatants (407 ng/μL) (Bonferroni, Scheffe, and Fisher LSD testing *F* = 52.23; *p* < 0.001). Moreover, the pellets revealed a predominance of dsDNA fractions over ssDNA and RNA and a predominance of RNA over a lower content of ssDNA in the given sample (Table 1B). The strongest correlated parameters in the overall Pearson’s analysis were between quantities of supernatants and pellets (Table 1C). The increase in TNA quality in the supernatant was accompanied by an increase in the TNA quality of pellets (r = 0.4; *p* < 0.001). Further comparison of the quantity and quality of TNA between the pellet and supernatant after centrifugation revealed differences in values for the period of investigation and grouping manner (Figure 2).

**Table 1:** Descriptive statistics for isolated and extracted TNA (A), variance analysis (B), and matrices of linear correlations (C).

(A)						
*N* = 80 samples	Pellet			Supernatant		
	TNA conc.	A260/280	A260/230	TNA conc.	A260/280	A260/230
	[ng/μL]	Absorbance		[ng/μL]	Absorbance	
Mean	51.39	2.24	1.42	407.21	2.27	1.97
Mean LCL 95%	47.23	2.22	1.37	384.25	2.27	1.94
Mean UCL 95%	55.55	2.26	1.47	430.17	2.28	2.00
Mean deviation	17.10	0.09	0.20	104.61	0.03	0.13
Mean standard error	2.10	0.01	0.02	11.61	0.00	0.01
Variance	609.49	0.01	0.07	18601.83	0.00	0.03
Coefficient of variation	0.48	0.05	0.19	0.33	0.02	0.09
Minimum	19.46	1.97	1.00	113.18	2.17	1.28
Maximum	185.10	2.67	2.46	805.60	2.40	2.30
Range	165.64	0.70	1.46	692.42	0.23	1.02
Sum*	7091.87			56194.98		
Median	46.72	2.25	1.40	389.05	2.29	2.00
Median error	0.22	0.00	0.00	1.24	0.00	0.00
IQR	24.95	0.13	0.30	169.55	0.05	0.22
Coefficient of dispersion	0.35	0.04	0.14	0.26	0.01	0.07
Skewness	2.28	0.50	1.31	0.71	−0.71	−1.06
Kurtosis	11.16	5.28	5.24	3.73	3.53	5.35

(B)														
Between	Difference		SE		t		df		P_Bonferroni_		P_Tukey_		P_Holm_	
	s	pl	s	pl	s	pl	s	pl	s	pl	s	pl	s	pl
Years 2024–2023	−66.2	−15.2	29.7	5.99	−2.23	−2.54	133		0.028	0.012	0.028	0.012	0.028	0.012
Methods 1–2	24.4	−4.45	28.3	5.7	0.862	−0.78			0.39	0.437	0.39	0.437	0.39	0.437
dsDNA-RNA	97.7	10.84	23.7	4.77	4.12	2.27			<0.001	0.074	<0.001	0.063	<0.001	0.049
dsDNA-ssDNA	159.9	19.92			6.75	4.18				<0.001		<0.001		<0.001
dsDNA-RNA	−62.2	−9.09			−2.63	−1.9			0.029	0.177	0.026	0.142	0.01	0.059

(C)					
Parameters		Q_TNA_pellet	A260/280	A260/230	Q_Supernatant
A260/230	r	0.267	0.317		
	*p*	0.002	<0.001		
Q_Supernatant	r	0.619		0.335	
	*p*	<0.001		<0.001	
A260/230_sup	r			0.408	0.477
	*p*			<0.001	<0.001

*The sum of the averages of the dsDNA, ssDNA, and RNA fractions for total period of investigation.

Q_TNA, the quantity of TNA.

s, supernatant; pl, pellet.

**Figure 2:**
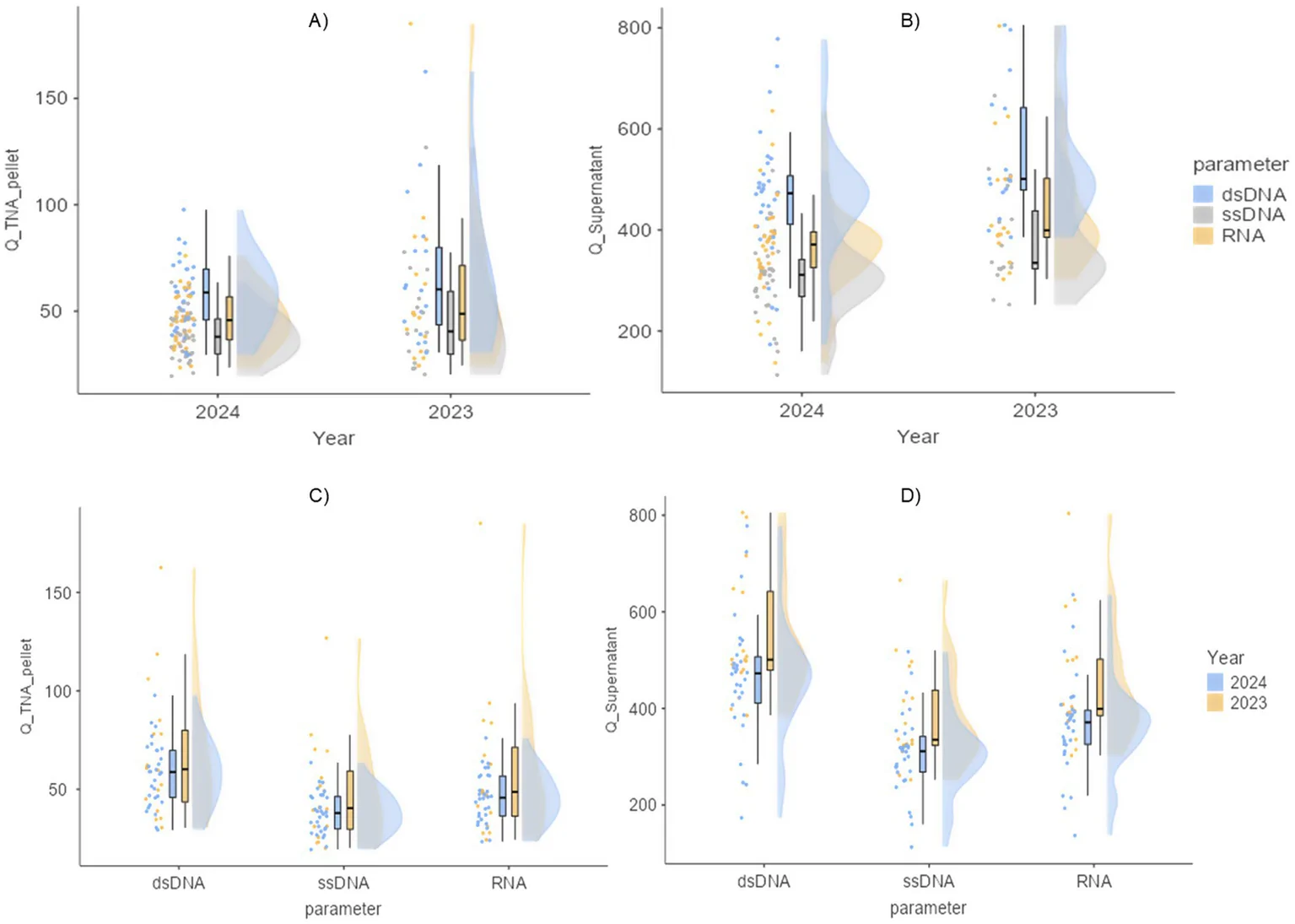
Raincloud plots of the quantity of TNA (ng/l) isolated from pellets **(A,C)** and supernatants **(B,D)**, grouped by time and parameters.

Considering the entire 20-month period of the study, pellet weight, the amount of isolated TNA, and its quality were positively correlated (Figure 3). Descriptive discriminant analysis (LDA) for all tested covariates revealed two main groups: collection point types and dependent variables (Figure 4A). According to Cohen’s and Hedges’ parameters, transportation collection points together with healthcare facilities were the strongest group (d(g) = +0.27) and housing estates with weaker WWTPs were the second group (d(g) = +0.14). Grouping covariates by TNA quality parameters separated the data into two (not distant) classes: dsDNA-RNA (d(g) = −0.42) and ssDNA-RNA (d(g) = +0.42) (Figure 4B).

**Figure 3:**
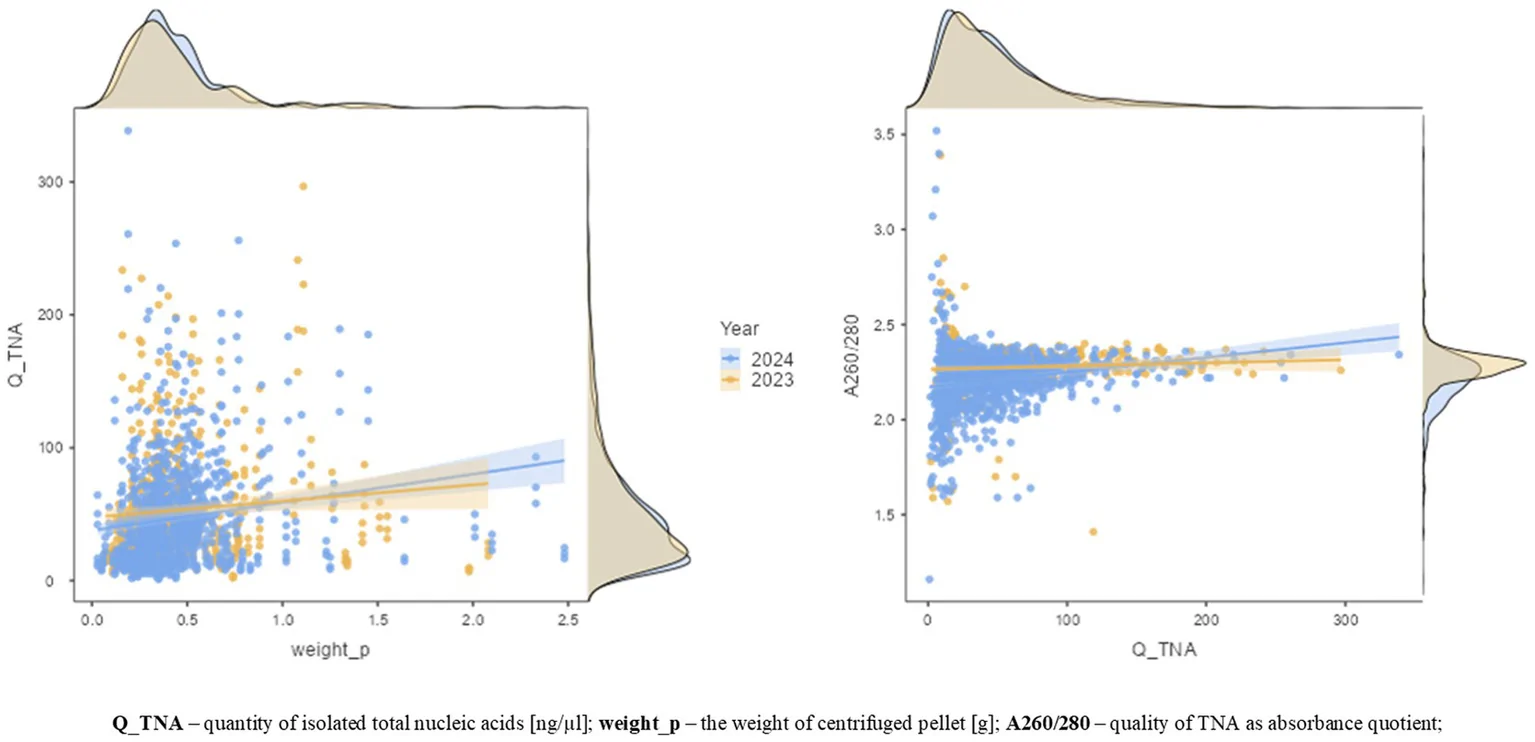
Pearson’s linear correlations (r) for all samples of pellet for all investigation periods. Q_TNA, quantity of isolated total nucleic acids [ng/ul]; weight_p, the weight of centrifuged pellet [g]; A260/280, quality of TNA as absorbance quotient.

**Figure 4:**
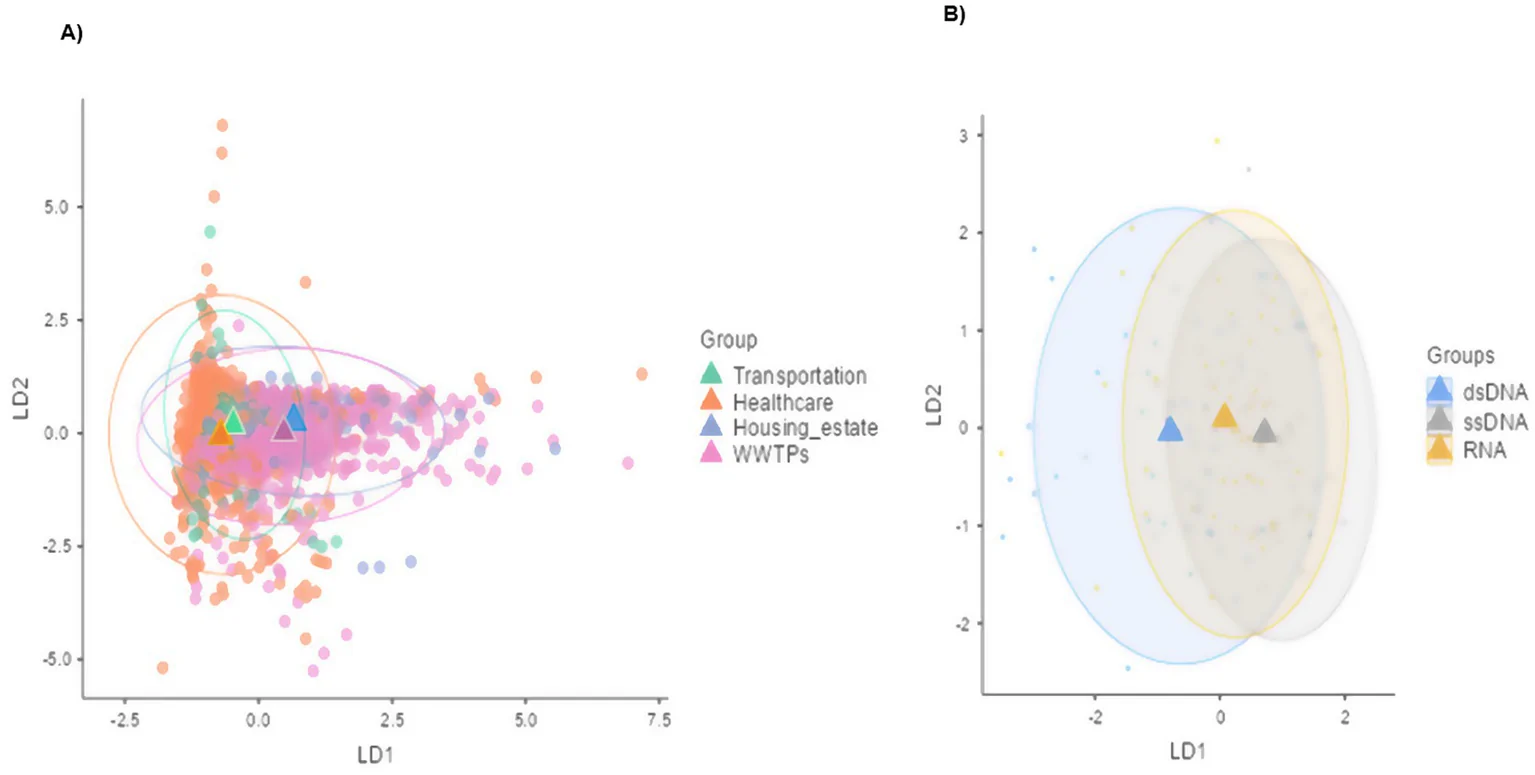
Descriptive discriminant analysis of the pellet weight, TNA quantity and quality as covariates, grouped by type of collection point **(A)** and parameter type **(B)** for the whole period of investigation.

### 3.3 The presence of molecular markers in the pellet and the supernatant

The marker selection in the analysis of SARS-CoV-2 virus RNA molecules in the catchment area included a comparative analysis of positive and negative RT-qPCRs based on differences in the resolution and specificity of E, N1, and N2 markers. Based on the positive-to-negative reactions ratio during preliminary selection, it was revealed that 20% of the samples were positive for E, 71% for N1, and 78% for N2 (Table 2). Prevalently, more virus copies were detected in supernatants than in pellets, with the exception of probe E. A comparison of probes N1 and N2 revealed the absence of amplification products in more than half of the pellets. Viral copy numbers in the pellet were an order of magnitude lower than in the supernatant for the N1 and N2 assays but three orders of magnitude higher for the E gene assay. Analysis of variance showed generally higher virus copy numbers obtained from the supernatant than from the pellet, except for E and N1, where the differences were not significant or the values were statistically identical (Figure 5). For probe N2, 85% of samples had a valid positive amplification result (Table 2, Figure 6A). In the case of probe N2, the number of copies amplified from the pellet grouped by collection sites in the catchment compared to supernatants did not differ significantly (Figure 6B). Detailed analysis of collection points indicated the most frequent absence of amplification products occurred in HCU samples in both sediments and supernatants. There was no correlation between the number of positive amplifications and the year or season of the analysis. For a given sampling site, valid amplification products were present in the sediment but absent in the supernatant. For probe N2, such situations were primarily observed in samples from hospitals in August 2023 and October 2024. From the pellets, 4,000 to 8,000 copies of sequences complementary to SARS-CoV-2 were recovered.

**Table 2:** Essential parameters during probe selection with special emphasis given to the supernatant and the pellet.

	% of positive reactions	Sum [c/μL]	Average [c/μL]	SD	*Post-hoc* one-way ANOVA
*n* = 40 (preselection)					
E overall	20	4.48E+06	1.15E+05	7.17E+05	S ≠ P *t = −0.05; p = 0.96*
Supernatant (S)	15	1.68E+03	8.86E+01	3.76E+02	
Pellet (P)	3	4.48E+06	2.24E+05	1.00E+06	
N1 overall	71	4.66E+03	1.16E+02	1.86E+02	S > P *t = 2.51; p = 0.02*
Supernatant	42	3.71E+03	1.85E+02	2.34E+02	
Pellet	30	9.50E+02	4.75E+01	7.79E+01	
N2 overall	78	2.25E+04	5.92E+02	1.09E+03	S ≠ P *t = −0.07; p = 0.94*
Supernatant	38	1.77E+04	8.86E+02	1.39E+03	
Pellet	40	4.78E+03	2.65E+02	4.57E+02	
*n* = 200 (2nd round)					
N1 overall	75	2.13E+06	1.66E+04	2.06E+02	S ≈ P *t = 0.73; p = 0.46*
Supernatant	100	1.45E+06	1.71E+04	2.17E+02	
Pellet	50	6.78E+05	1.58E+04	1.77E+02	
N2 overall	71	5.43E+06	4.34E+04	2.15E+03	S ≠ P *t = 0.97; p = 0.33*
Supernatant	100	4.98E+06	5.66E+04	5.13E+02	
Pellet	42	4.48E+05	1.21E+04	9.21E+03	
*n* = 934 (final application)					
N2 overall	80	3.04E+08	1.75E+05	3.70E+05	S > P *t = −13.0; p < 0.001*
Supernatant	95	2.86E+08	3.27E+05	4.76E+05	
Pellet	85	1.71E+07	2.25E+04	4.85E+04	

*Post-hoc* tests for Tukey, Scheffe, Bonferroni and Holm; [c/μL]—the number of virus copies per microliter.

**Figure 5:**
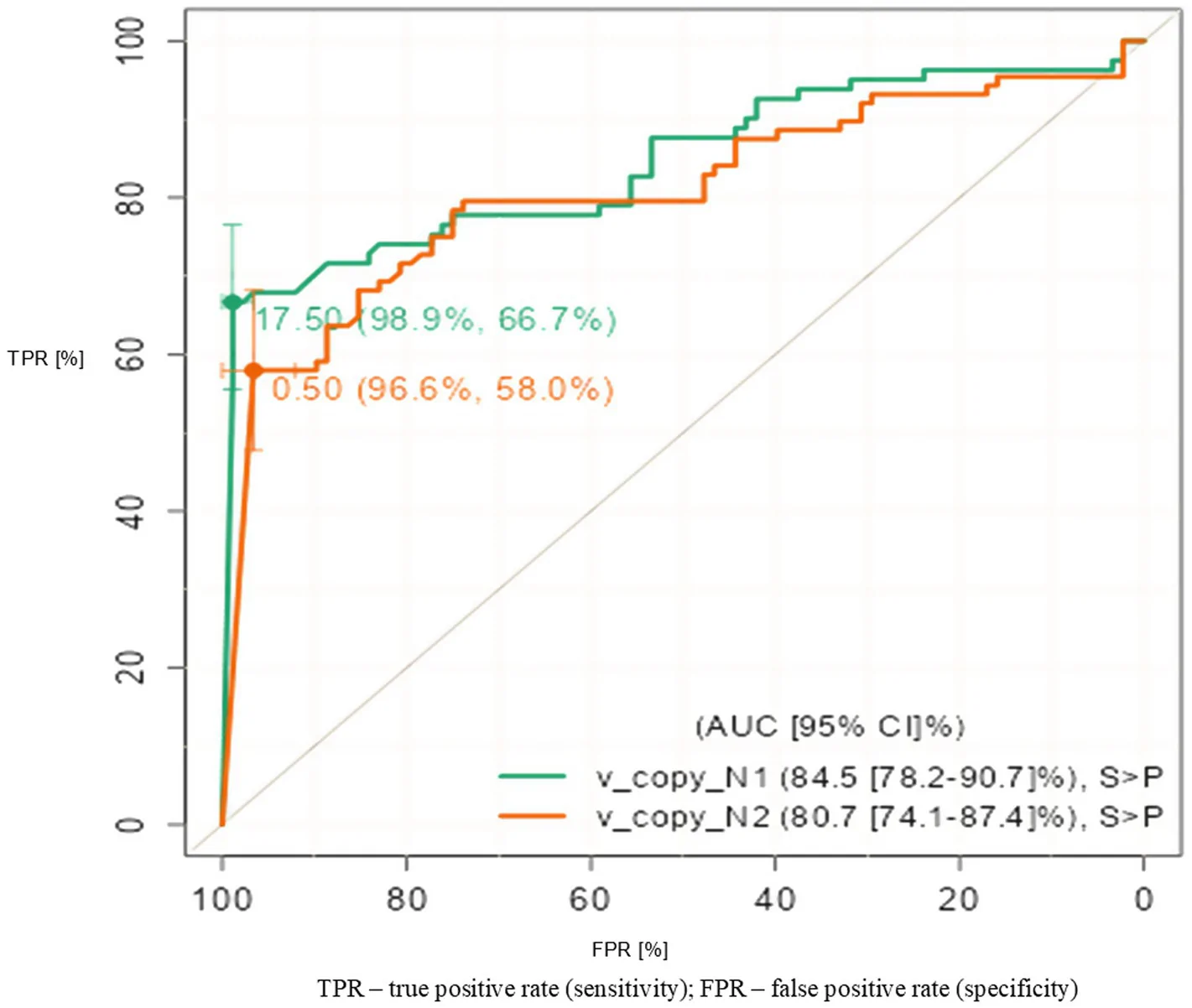
Receiver operating characteristic curves for virus copies as predictors, classified for supernatant (S) and pellet (P) phases, and grouped by N2 probe.

**Figure 6:**
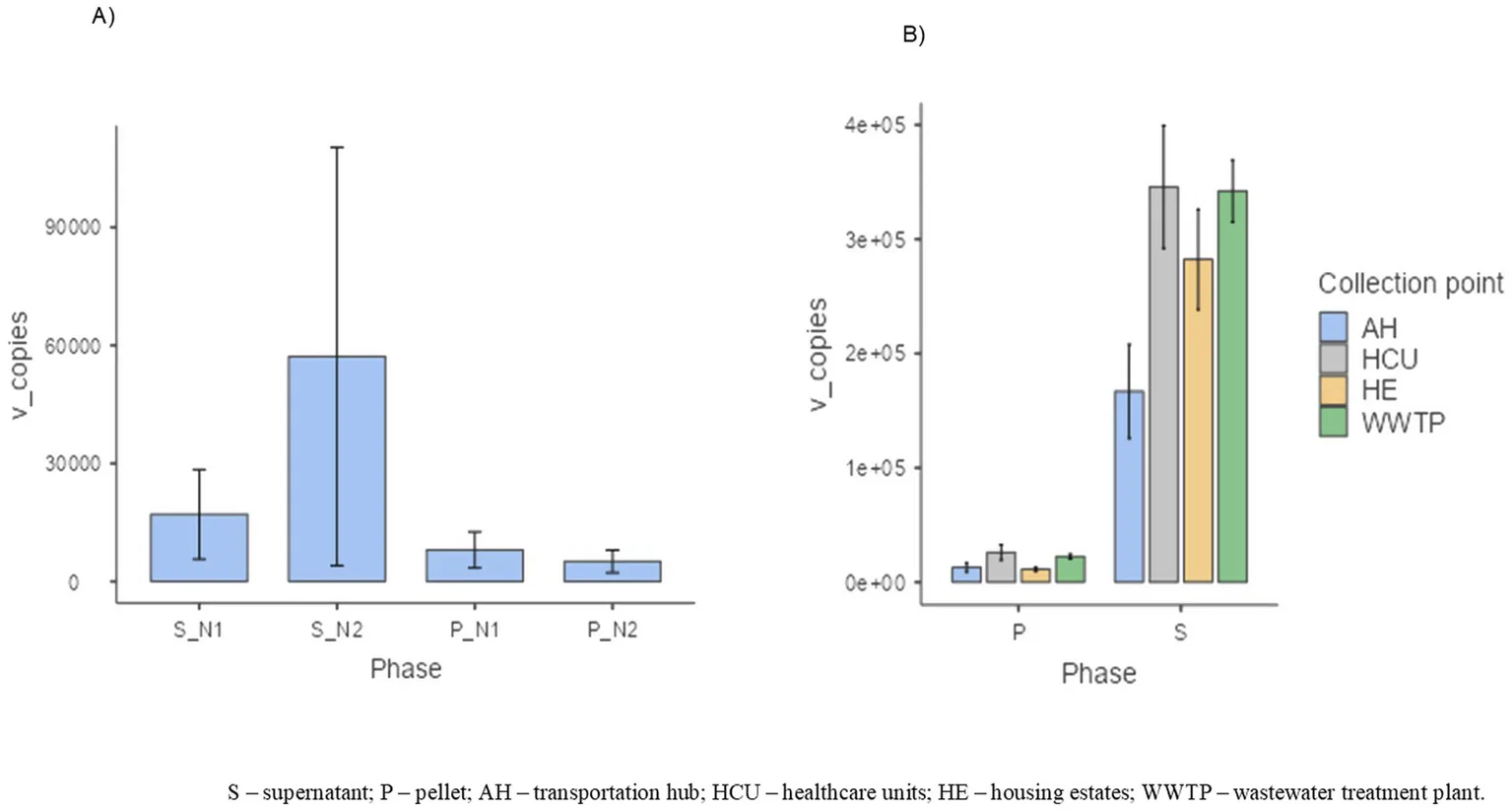
The number of SARS-CoV-2 copies per microliter of amplification mix divided by phases after centrifugation for N1/N2 probes **(A)** and for N2 probe with grouping according to type of collection point **(B)**. S, supernatant; P, pellet; AH, transportation hub; HCU, healthcare units; HE, housing estates; WWTP, wastewater treatment plant.

The molecular probes used in the analysis of the quality of the sewage catchment with bacteria as the biomarker also showed variable occurrence in the supernatant and the pellet for individual sewage collection points (Table 3). In a sample pool of 72 PCR reactions for the pellets, the number of positive reactions ranged from 5 (for MRSA) to 70 (for Verona Integron Methicillinase strains, VIM). One-way ANOVA and post-hoc tests revealed no differences in the frequencies of positive qPCR results in the pellets between methods, years, and months. A higher frequency of positive reactions was observed in wastewater treatment plant pellets compared to transportation hubs (t = −2.9; *p* = 0.022). Generally, positive results were obtained less frequently in pellets than in supernatants, except for reactions for MSSA and met-oxa-R strains. Moreover, the frequency of qPCR reaction product for bacterial and chemotherapeutic resistance probes in pellets was positively correlated with that in the supernatant (Pearson r = 0.60; *p* < 0.001). Similarly, as in the case of virus surveillance, for bacteria, for a given sampling site, the presence of valid amplification products in the sediment was demonstrated, while their absence was observed in the supernatant (Supplementary Table 4). In total, in 72 collection points, there were 46 cases of a positive reaction in pellets and a negative reaction in supernatant, most often for health care units and transportation hubs. This was especially true for enteropathogenic *Escherichia coli* strains and methicillin susceptible *Staphylococcus aureus* (Supplementary Table 4, Figure 7). Moreover, the MSSA marker was more frequent in HE pellets than in WTP, and for MRCoNS this frequency was higher in WTP pellets than in HE and HCA samples (*p* < 0.05). Pearson’s correlation analysis revealed the highest values between the frequency of positive results in the pellet for VIM and STEC markers (r = 1.00; *p* < 0.001) and *Shigella* sp. type 1 and EHEC markers (r = 0.81; *p* < 0.001).

**Table 3:** Frequency of presence for positive qPCR reactions for bacterial probes with division by the supernatant and the pellet.

	Phase of the sample	Positive reactions	Average frequency	SD	SE	t_Tukey*_	*p*
Enterohemorrhagic *Escherichia coli* (EHEC)	Supernatant	48	0.6667	0.475	0.0559	3.83	0.001
	Pellet	26	0.3611	0.484	0.0570		
Shiga toxin *Escherichia coli* (STEC)	Supernatant	18	0.2500	0.436	0.0514	2.73	0.007
	Pellet	6	0.0833	0.278	0.0328		
*Shigella* type1	Supernatant	26	0.3611	0.484	0.0570	2.47	0.015
	Pellet	13	0.1806	0.387	0.0456		
Vancomycin resistance gene (*vanA*)	Supernatant	58	0.8056	0.399	0.0470	3.32	0.001
	Pellet	40	0.5556	0.500	0.0590		
Vancomycin resistance gene (*vanB*)	Supernatant	72	1.0000	0.000	0.0000	2.30	0.023
	Pellet	67	0.9306	0.256	0.0302		
New Delhi metallo-β-lactamase bacteria (NDM-1)	Supernatant	56	0.7778	0.419	0.0493	2.19	0.030
	Pellet	44	0.6111	0.491	0.0579		
*Klebsiella pneumoniae* carbapenemase (KPC)	Supernatant	72	1.0000	0.000	0.0000	2.54	0.012
	Pellet	66	0.9167	0.278	0.0328		
*Salmonella* sp.	Supernatant	28	0.3889	0.491	0.0579	2.19	0.030
	Pellet	16	0.2222	0.419	0.0493		
Methicillin resistant *Staphylococcus aureus* (MRSA)	Supernatant	21	0.2917	0.458	0.0539	3.60	0.001
	Pellet	5	0.0694	0.256	0.0302		
Methicillin susceptible *Staphylococcus aureus* (MSSA)	Supernatant	9	0.1250	0.333	0.0392	−2.87	0,005
	Pellet	23	0.3194	0.470	0.0553		
Methicillin and oxacillin resistant other than *Staphyloccoccus aureus* (met-oxa-R)	Supernatant	6	0.0833	0.278	0.0328	−2.14	0,034
	Pellet	15	0.2083	0.409	0.0482		

*Post-hoc Tukey test for df 142.

**Figure 7:**
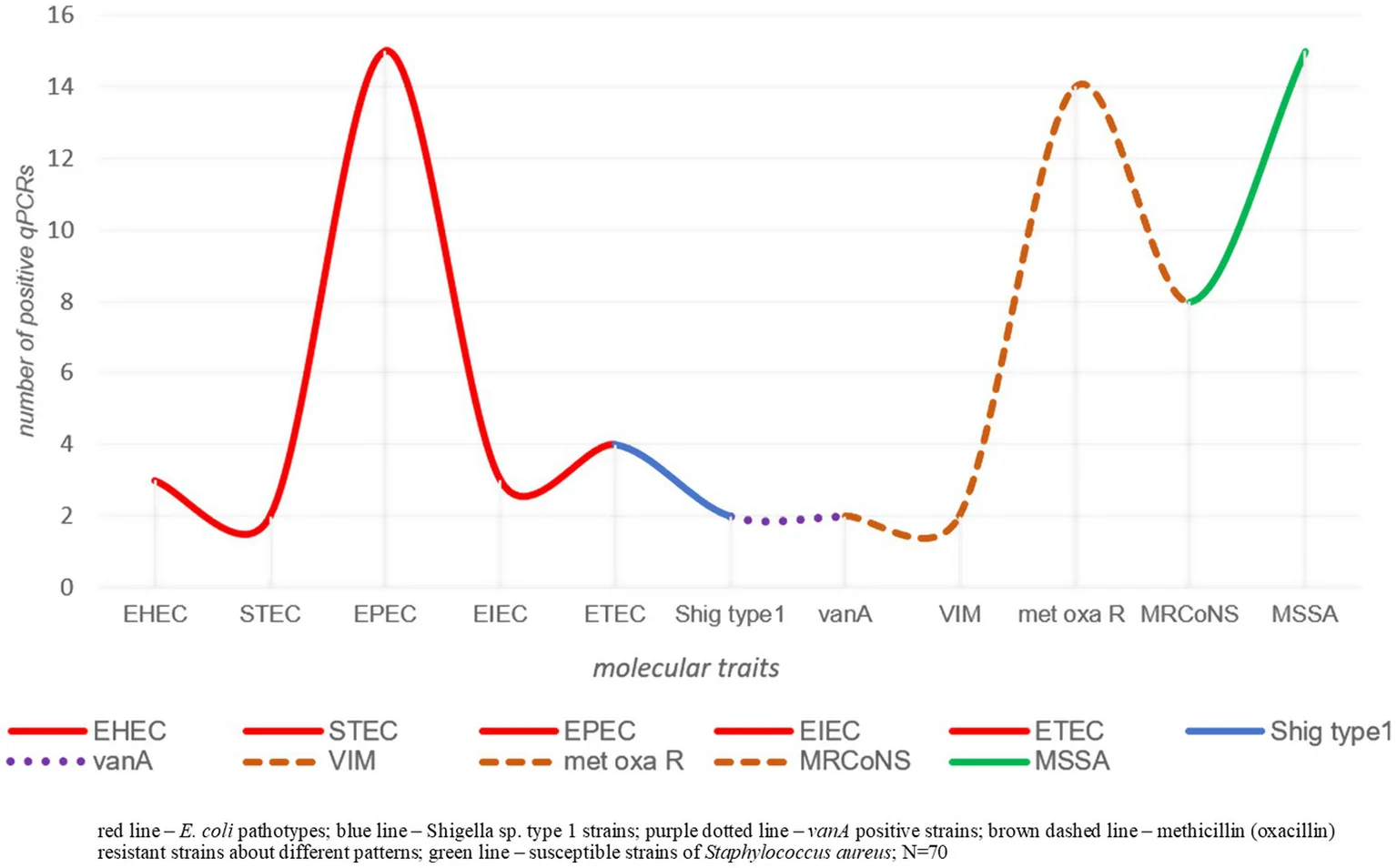
The number of positive qPCRs in pellets and simultaneous negative results in supematants for applied molecular markers during qualitative scanning of municipal sewage catchment. Red line- *E. coli* pathotypes; blue line- Shigella sp. type 1 strains; purple dotted line -vanA positive strains; brown dashed line-methicillin (oxacillin) resistant strains about different patterns; green line - susceptible strains of *Staphylococcus aureus*; *N* = 70.

## 4 Discussion

During routine sanitary and hygienic surveillance of the Warsaw municipal sewage catchment area, total nucleic acid (TNA) was isolated, separated, and purified. The genetic information contained in TNA served to qualitatively and quantitatively assess the number of SARS-CoV-2 virus copies, as well as qualitatively assess the presence of re-emerging, ESKAPE pathogens and the presence of selected chemotherapeutic resistance traits. All these activities began with the process of physical concentration, including vacuum and gravity (10). This study focused primarily on the quantitative and qualitative characteristics of the separated phases, specifically whether target genetic material in the pellets significantly influenced analytical outcomes or had a negligible impact (11). Typically, research procedures focus on obtaining a clear supernatant for use in subsequent research steps. However, the direct cause of the pellet formation in samples is both organic and inorganic particles in raw sewage and activated sludge residues in treated sewage (12).

The first verified hypothesis (H1) is false, and there are no significant differences between the weight of the pellets for periods and collection points during the presented investigation, except for three analytical series in July 2023 (July 5, 12, and 18). The weight of the pellets obtained on these days were lower than those of seven other research series within the 18-month period (Supplementary Tables 1A,B, 2 and Supplementary Figures 1A–F). The low level of weight variability of sediments (sludge) in July and the high variability in December were caused by low rainfall, slower sewage velocity in collectors, higher sedimentation rate in net sewage and, in winter, high precipitation and higher loads of sewage discharged from housing estates. The higher variability and high sediment weights in winter samples also reflect the significant impact of the lockdown (COVID-19) on a society largely confined to activities in their homes. The obtained correlations of the sediment weight for the summer and autumn samples seem to be random, and the inverse Pearson correlations in the summer of two consecutive years could be due to the phasing out of the lockdown in the country (2024) and the increase in the qualitative and quantitative parameters of sewage in the catchment. Moreover, during the study period, the sample color, turbidity, and odor did not differ from those commonly described in the literature. However, in the case of hospital wastewater, there have been cases of milky, lemony, or dark tea-like liquids with a strong chemical or putrid odor (13). It appears valuable to perform chromatographic studies to determine the composition of these samples. However, these specific wastewater samples did not appear to have a direct impact on the subsequent amplification reaction results (14, 15).

The second verified hypothesis (H2) is true, and the weight of the pellet was proportional to the concentration of TNA obtained after extraction, isolation, and cleaning. Moreover, the increase in the amount of sediment fraction in the samples was consistent with an increase in the quality measured by the absorbance quotient A260 and A280 nm (16). The sediments were also most abundant in double-stranded DNA and RNA fractions and somewhat prevalent in single-stranded DNA. Oligonucleotide fractions were also present in the pellets, constituting the smallest percentage of genetic material of the lowest quality.

The third verified hypothesis (H3) is true, and the quantity and quality of total nucleic acid (TNA) isolated from sediment samples differed from the quality of TNA isolated from the aqueous phase for each sample tested. The TNA isolated from the sediments was one-eighth of the amount recovered from the supernatants. The quality of TNA measured as the ratio A260/280 nm isolated from sediments was significantly lower than in supernatant (ANOVA, F Durbin-Conover = 4.09; *p* < 0.01), as was the quality measured by the A260/A230 parameter. However, the average TNA quality in the sediments was still very high (2.24), and the differences in the quality of nucleic acids between samples of the pellet did not exceed 0.04. This means that the pellet samples contained significantly less high-quality TNA than the TNA samples isolated from the supernatant, while exhibiting minimal qualitative and quantitative differences within the respective group of pellet or supernatant samples (intra-group homogeneity). The higher proportion of proteins in the pellet was not that noticeable and did not affect the subsequent quality of the analyses. The variation between the quality of the pellet samples relative to the samples of aqueous phase measured by the A260/A230 (average 1.42 for pellet versus 1.97 for supernatant) parameter is explained by the stronger binding of residual solvents and isolation reagents with organic particles and inorganic substances of sewage sludge during TNA isolation. Residual guanidine is known as a common disturbing factor during column-based kit isolation and, in our research, it resulted in an overestimation of the nucleic acid concentration. Samples with low values of this parameter occasionally contained three to four orders of magnitude more copies of the SARS-CoV-2 virus in the pellet than in the supernatant or resulted in inconclusive results during bacterial surveillance over the catchment. Such samples were analyzed repeatedly and often omitted in further stages of research.

The fourth verified hypothesis (H4) is true, and the number of positive reactions differed in the pellet and the supernatant after the amplification. This relationship served as a selection of molecular probes during surveillance of the catchment during COVID-19 in Poland. Among the three recommended primer–probe sets (E, N1, and N2), one was selected based on its performance in wastewater matrices, including analytical specificity, sensitivity, and amplification efficiency (17). This response was variable between and among probes, both within and between the groups of post-centrifugation phases tested. The probe complementary to the virus envelope genes (E) gave unstable results, more often showing the presence of the virus only in the supernatant (Table 2) (18). At the same time, the total number of particles suggested a much higher number of viruses in the sediments. Further analysis did not yield convincing results compared to other probes tested simultaneously, as they were often contradictory, and ultimately, probe E was omitted from further studies as it was highly unstable. Preselected N1 and N2 probes showed either significantly more RT-qPCR products (N1) or negligible differences (N2) between the pellet and supernatant matrices (Table 2, Figures 5, 6) (19). In a further increased number of samples, the N1 probe showed no differences in the number of copies detected in the post-centrifugation phases and was omitted from further analysis. An additional indicator for omitting the N1 sequence was the higher total load of SARS-CoV-2 particles detected in the sediments than in the aqueous phase of the samples. Ultimately, the number of SARS-CoV-2 virus copies was determined using N2 as a molecular marker of COVID-19 dynamics in the capital’s catchment area, which yielded satisfactory methodological, statistical, and functional results on a sample of nearly 1,000. Moreover, results revealed a significantly lower number of virus-complementary molecules in sediment samples than in supernatants. Nevertheless, there was still a high percentage of positive results and high values of virus particles detected in the pellets. Interestingly, at some sampling sites, valid amplification products occurred in the pellet but were absent in the supernatant. Rejecting information contained in the pellet for samples collected from hospitals (August 2023 and October 2024) would make sampling points invisible in the pool of statistical results and reduce the number of COVID-19 cases obtained from transportation hubs and hospitals (H5 = true).

Analyses of re-emerging pathogen circulation, ESKAPE pathogens, and chemotherapeutic resistance markers further supported Hypothesis 4, in agreement with Smith et al. (20). Compared to virus surveillance using the RT-qPCR technique, qualitative studies of prokaryotic pathogens revealed more pronounced differences between matrix responses in the phases. Nine of the twenty-one molecular probe groups used showed a significantly lower frequency of qPCR amplification products in the pellet compared to the supernatant. In the case of probes identifying methicillin-sensitive *Staphylococcus aureus* strains (MSSA) and those possessing the *mecA* and *mecC* genes, without the confirmed presence of the SA442/CoA element (other than *S. aureus*), positive reactions were obtained significantly more often in precipitates than in aqueous phases (Table 3, Supplementary Table 4, Figure 7). At the same time, probes identifying methicillin-resistant Verona-type strains, *Shigella* sp. type 1, and enterohemorrhagic *E. coli* (EHEC) strains showed positive correlations with each other in the pellets (21). The results of qualitative reactions using eleven pre-selected sets of molecular probes in the analysis of the occurrence of prokaryotic pathogens also revealed the frequent occurrence of valid positive results in the pellets and their simultaneous absence in the supernatant. These differences were more pronounced and frequent than in the SARS-CoV-2 studies. The reason for this phenomenon may be the different composition of the reaction mixtures and the higher specific gravity of bacterial cells and cell wall remnants, which under the influence of centrifugation gravity (g force) could incorporate, disturb, or otherwise ‘pull’ the genomic DNA of bacteria and parts of the sequence towards the pellets (22). In this study, this phenomenon was particularly frequent in the case of enteropathogenic *E. coli* (EPEC) strains, methicillin- and oxacillin-resistant strains, and methicillin-sensitive *Staphylococcus aureus* strains. If the results obtained from sediments were omitted, the loss of information on the presence of all *E. coli* pathotypes would distort the picture of their circulation for all catchment points of the capital in the periods from May to August 2023 and from September to December 2024 (H5 = true). Another loss would be a distorted qualitative picture of met-oxa-R and MSSA strains in wastewater from all healthcare facilities in the capital, in the period from June 2023 to September 2024 (H5 = true). It would also be impossible to draw conclusions about the quality of wastewater in the period February–December 2024 in terms of the presence of methicillin-resistant, coagulase-negative streptococci (H5 = true). The above results can be confirmed by numerous similar studies, including Desneux and Pourcher (23), Gryp et al. (24), Liu et al. (25), and Fofiu et al. (26).

The methodological aspect of this study was also to quickly check the possible impact of changes in ethanol concentration from 95 to 96% and centrifugation conditions from ‘room temperature’ to a stabilized +18 °C. The first aspect was the more widespread availability of 96% alcohol, including the avoidance of possible dilutions and the associated variability errors. The second aspect was related to the difficulty of maintaining stable temperature conditions in the room and the device. Temperature variations in the initial phases of the research at the centrifuge station ranged between +20 °C and +26 °C, depending on the intensity of work, time of day, and year. Remembering the increase in sedimentation efficiency by centrifugation at lower temperatures, +18 °C was considered a convenient compromise. It turned out that the obtained results did not differ significantly in years or months of adjacent years or changed methodology (27). This methodological goal was undertaken due to the adsorption of particles on suspended solid elements present in wastewater. Tao et al. (28) demonstrated that EVs readily adsorb small solid particles suspended in sewage. They indicated that the number of virions recovered from the solids even exceeded that recovered from the supernatant during river investigation, similar to what we investigated. At the same time, these authors pointed out the ambiguity about whether different serotypes have a similar propensity to adsorb the solids (28). In our studies, we did not work with different genotypes and variants of SARS-CoV-2; however, we showed that enteropathogenic *E. coli* (EPEC) and methicillin-sensitive *Staphylococcus aureus* are more common in sediments than in the supernatant after wastewater centrifugation (Supplementary Table 4). Taking into account the adsorption of virions, bacterial cells, and TNA elements on suspended solids in sewage is undoubtedly an important step towards a reliable assessment of the excretion rate of these particles in the sewage system (10, 29). The production of mucous capsules (adhesins), the agglomeration capacity of cocci and rods, the predisposition to take up short DNA fragments by *Pseudomonas*, the release of spores by disrupted bacilli, and even the phenomenon of quorum-sensing all increase the intensity of adhesion or precipitation to sewage pellet during or after centrifugation (30). As wastewater is a complex sample matrix, it contains numerous qPCR inhibitors, the concentration and diversity of which depend on population size, type of industry and agriculture, and climate. Differences in the qPCR inhibitor profile can affect data quality, potentially leading to erroneous results (14). Fully aware of the importance of this problem, we have not undertaken a detailed analysis of the type, species, number, and effects of qPCR inhibitors due to economic considerations. However, basic physicochemical observations of raw sewage, such as variation in color, odor, and pH, did not reveal statistically significant differences in the results after amplification across runs and replicates from a given sampling site and over time. Physicochemical changes were not correlated with the variable number of ‘inconclusive’ reactions. All RT-qPCR and qPCR reactions were valid.

The economic aspect of the presented research consisted of costs, laboratory effort, and gain in the sensitivity of results according to the most probable number of tested bio-factors. All analyses were performed by a single technician with a strong scientific and research background, with support from three staff as needed. The main technician and one technologist at the Wastewater Treatment Plant were responsible for organizing the research. Therefore, during the study period, a single full-time position was utilized, with an expanded workload of 10 h, 3 to 4 days a week. Dedicated kits for full-scale SARS-CoV-2 analysis were used without unnecessary cost savings, as well as available IVD tests for qualitative and quantitative analysis of bacteria and chemotherapeutic resistance genes. During the analysis, the economic mechanisms of the supplier market forced cost savings and the identification of a single marker for SARS-CoV-2. The deteriorating geopolitical situation in Eastern Europe also adversely affected the market and prices of IVD qPCR tests for selected bacteria and genes. Rising prices and supply constraints forced optimization of qPCR tests to increase their efficiency. The comparative results of these optimizations are promising and allow for a reduction in analysis costs by half (unpublished data). In summary, the cost of a complete (proposed) analysis of a single sample of sludge and supernatant after sewage centrifugation is between 100 and 170 euros. These calculations do not include equipment depreciation, utility costs, or other indirect costs. The options we chose guaranteed appropriate quality at a price. The above calculations provide a very general view of analysis costs and have decreased after the end of the COVID-19 pandemic. The economic aspect, although important, will not determine requirements but only regulate the possibilities in this regard.

## 5 Conclusion

In the course of the presented research, five basic hypotheses were verified, the first of which turned out to be false and the other four true. Firstly, the method for TNA isolation from municipal wastewater in a large agglomeration was optimized at the initial stage, with separation and characterization of the obtained phases in accordance with available market procedures (kits). Therefore, the suitability of the pre-formulated kits for the isolation of TNA from wastewater for the purposes of pandemic monitoring and sanitary surveillance over a two million population equivalent was confirmed. Second, despite yielding a higher quantity of TNA due to larger pellet volume, this material exhibited lower quality than TNA recovered from the supernatant. Third, conducting tests on both the supernatant and the pellet after centrifugation will prevent underestimation (loss) of qualitative and quantitative data, and the ‘net gain’ in this sense may be the detection of the factor or a significantly higher number of the bioindicator. Undoubtedly, in the context of similar studies, it is valuable to investigate the effects of the many inhibitors present in wastewater, as it is a very complex matrix. Clear guidelines for solid- and liquid-phase wastewater analyses for anti-epidemic purposes will facilitate the interpretation of results from many different research centers. Above all, however, it can significantly eliminate manipulation of results and misinformation provided to the medical services during a crisis. The research results constitute a filling of the gap for one of many methodological aspects indicated by Article 17 of the “Urban wastewater surveillance” Directive (EU) 2024/3019 of the European Parliament and of the Council for all sections of the Directive. The idea and proposal for microbiological bioindicators and determinants of chemotherapeutic resistance should be included in the catalogue of environmental diagnostic tests during an epidemiological crisis, as we suggested and tested methodologically.

Taking all evaluated factors into account, the trade-offs are clear. Failure to perform sediment testing may result in a loss of information about the presence of microorganisms and drug resistance markers and failure to implement appropriate preventive procedures. Lack of information from sediments may also contribute to final reporting of fewer cases of infection, which may result in the implementation of insufficient countermeasures.

## Data Availability

The raw data supporting the conclusions of this article will be made available by the authors, without undue reservation.
