# Supplementary material for: Microbiological information in the precipitate and supernatant fractions after wastewater centrifugation—loss or profit for hygiene, epidemiology, and environmental surveillance?
Source: Front Public Health. 2026 Oct 26;14:1897003. doi: 10.3389/fpubh.2026.1897003 (PMC13745672; doi:10.3389/fpubh.2026.1897003)
Supplement: Supplementary file 1 [file Data_Sheet_1.PDF]

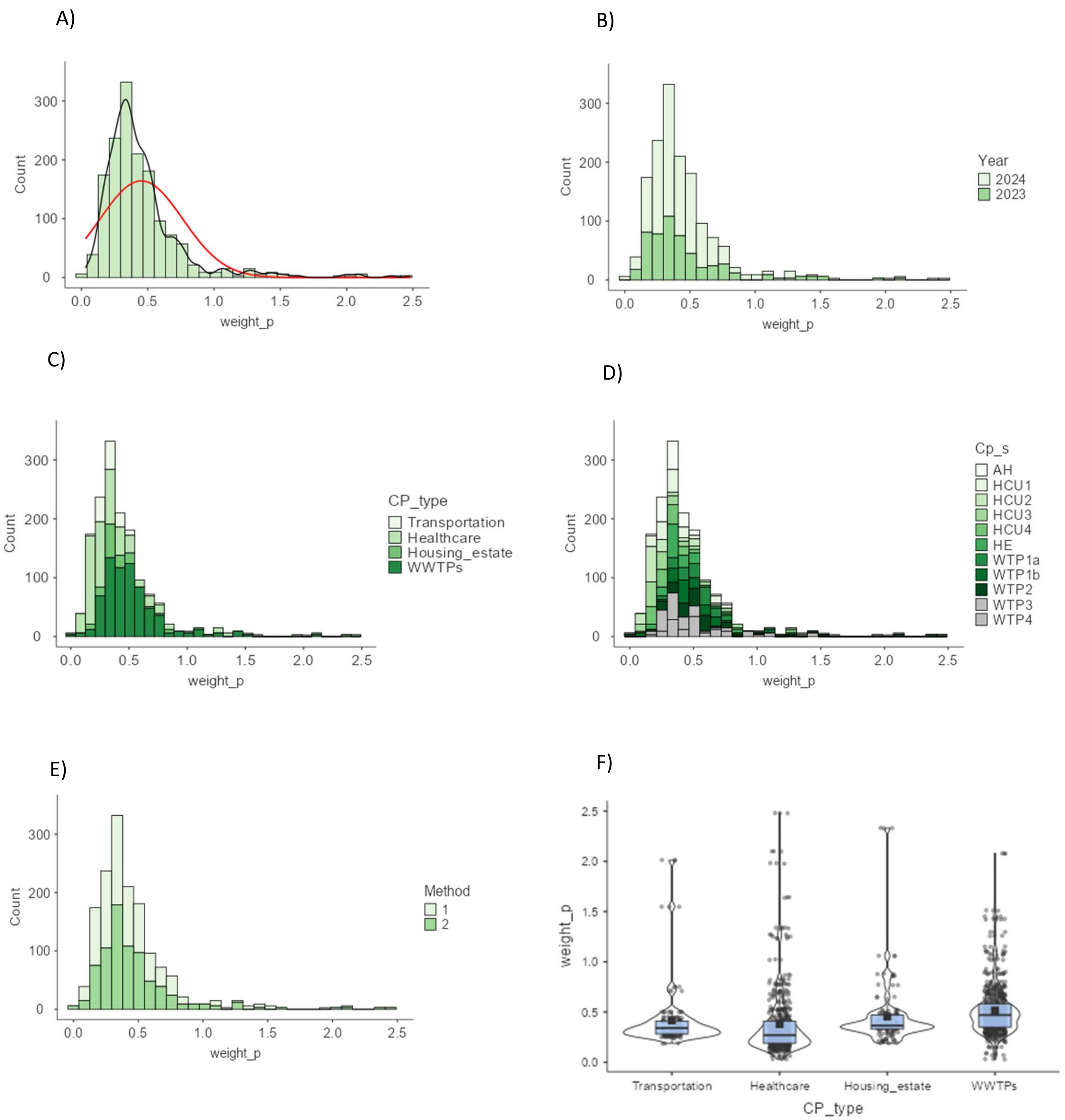

weight_p – pellet weight after centrifugation; **black line** – density; **red line** – normal curve; Cp_type - classification of catchments; Cp_s - marking sampling points in catchments; method 1 – classical extraction method, according manufacturer; method 2 – modified method with centrifugation (5000/10min/18°C) and 95% EtOH; F) – box-violine like plot with random biases and average values.

AH – transportation hubs; **HCU** – healthcare units and facilities;
HE – housing estates; **WTP** - wastewater treatment plants' inlets;

Supplementary Fig. 1. Pellet weights [g] after centrifugation: A) grouped by years, B) collection type on catchment C), detailed collection points D), method of TNA isolation E), with elements of descriptive statistics on violin-box plot, mean values and random bias of data grouped by general type of collection point, presented on cumulative bar charts F).

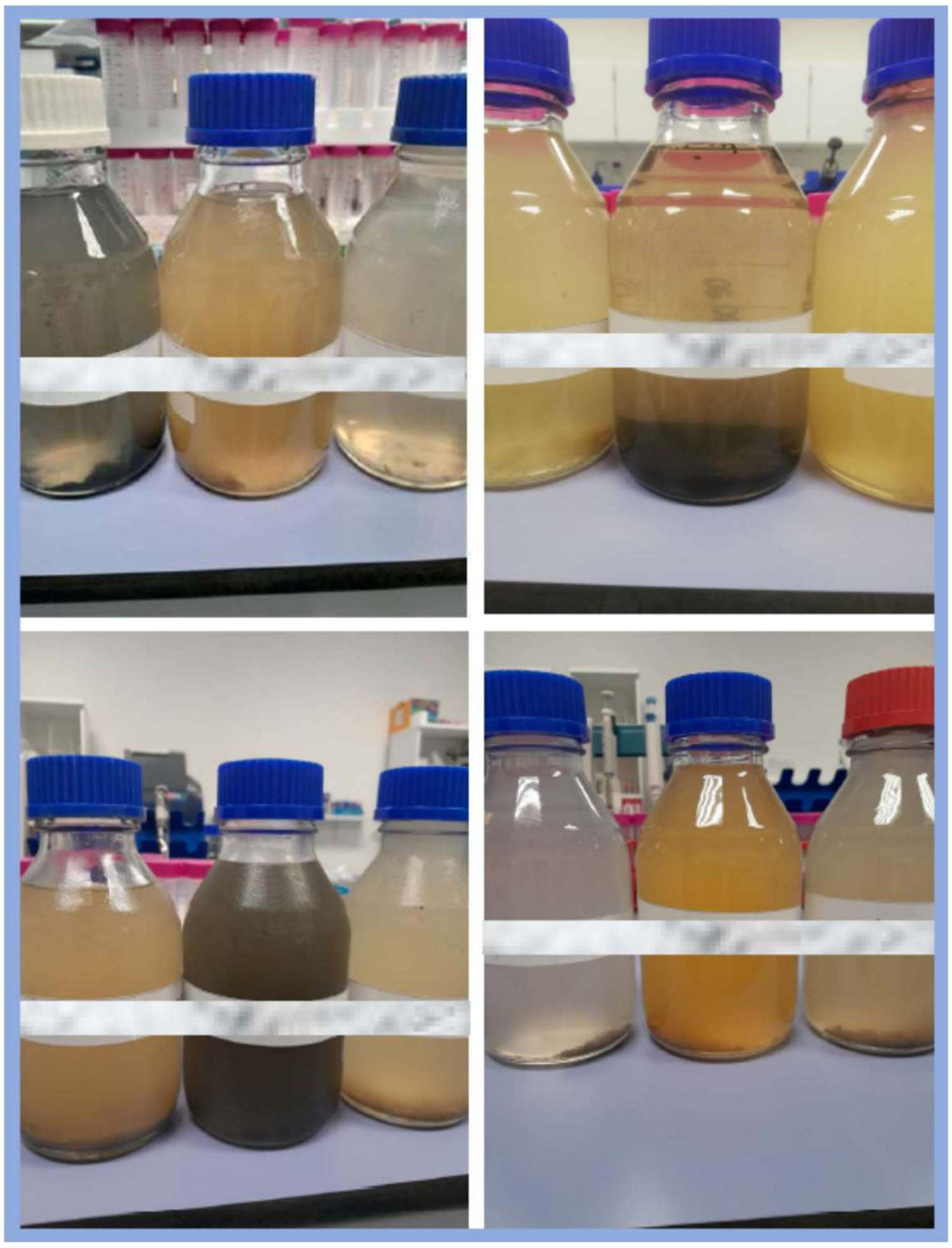

Supplementary Fig. 2. Samples before or after preliminary sedimentation in bottles from 12 different collection points and days varied in the volume and colour of the sediments and suspended solids.
