# Supplementary material for: Microbiological information in the precipitate and supernatant fractions after wastewater centrifugation—loss or profit for hygiene, epidemiology, and environmental surveillance?
Source: Front Public Health. 2026 Oct 26;14:1897003. doi: 10.3389/fpubh.2026.1897003 (PMC13745672; doi:10.3389/fpubh.2026.1897003)
Supplement: Supplementary file 2 [file Supplementary_file_1.DOCX]

**Supplementary Table 1.** Descriptive statistics for centrifuged pellets in 2023 A) and 2024 B).

**A)**

| DATE | Mean  [g] | SE | LCL90% | UCL90% | Median | Sum [g] | SD | Variance | IQR | Range  [g] | Min.  [g] | Max. [g] | Skewness | Kurtosis | t-student* | p* | W** | p** |
| --- | --- | --- | --- | --- | --- | --- | --- | --- | --- | --- | --- | --- | --- | --- | --- | --- | --- | --- |
| 30.06.2023 | 0.22 | 0.04 | 0.13 | 0.31 | 0.20 | 2.42 | 0.13 | 0.02 | 0.19 | 0.39 | 0.06 | 0.45 | 0.43 | -0.90 | 4.11 | 0.03 | 0.91 | 0.46 |
| 05.07.2023 | 0.49 | 0.08 | 0.30 | 0.67 | 0.48 | 5.34 | 0.27 | 0.07 | 0.32 | 0.97 | 0.06 | 1.03 | 0.51 | 0.33 | 2.80 | 0.07 | 0.90 | 0.43 |
| 12.07.2023 | 0.20 | 0.05 | 0.09 | 0.30 | 0.12 | 2.16 | 0.16 | 0.02 | 0.21 | 0.47 | 0.02 | 0.49 | 0.75 | -0.71 | 2.05 | 0.13 | 0.87 | 0.30 |
| 18.07.2023 | 0.16 | 0.04 | 0.06 | 0.26 | 0.14 | 1.58 | 0.14 | 0.02 | 0.14 | 0.41 | 0.01 | 0.42 | 0.91 | -0.06 | 2.74 | 0.07 | 0.95 | 0.71 |
| 26.07.2023 | 0.44 | 0.08 | 0.27 | 0.61 | 0.35 | 4.84 | 0.25 | 0.06 | 0.25 | 0.83 | 0.18 | 1.01 | 1.38 | 1.59 | 3.73 | 0.03 | 0.80 | 0.10 |
| 02.08.2023 | 0.66 | 0.12 | 0.39 | 0.93 | 0.51 | 7.30 | 0.40 | 0.16 | 0.61 | 1.31 | 0.13 | 1.44 | 0.67 | -0.45 | 2.64 | 0.08 | 0.93 | 0.57 |
| 09.08.2023 | 0.49 | 0.10 | 0.28 | 0.70 | 0.36 | 5.37 | 0.32 | 0.10 | 0.17 | 1.08 | 0.16 | 1.24 | 1.70 | 2.59 | 4.36 | 0.02 | 0.94 | 0.67 |
| 23.08.2023 | 0.51 | 0.08 | 0.34 | 0.68 | 0.47 | 5.57 | 0.25 | 0.06 | 0.16 | 0.90 | 0.21 | 1.11 | 1.49 | 2.82 | 4.04 | 0.03 | 0.90 | 0.41 |
| 30.08.2023 | 0.36 | 0.03 | 0.28 | 0.43 | 0.35 | 3.95 | 0.11 | 0.01 | 0.08 | 0.43 | 0.16 | 0.59 | 0.37 | 1.59 | 15.35 | <.001 | 0.98 | 0.93 |
| 06.09.2023 | 0.39 | 0.06 | 0.25 | 0.51 | 0.37 | 4.23 | 0.19 | 0.04 | 0.21 | 0.69 | 0.11 | 0.80 | 0.78 | 0.94 | 5.90 | 0.01 | 0.98 | 0.90 |
| 13.09.2023 | 0.39 | 0.06 | 0.25 | 0.51 | 0.37 | 4.23 | 0.19 | 0.04 | 0.21 | 0.69 | 0.11 | 0.80 | 0.78 | 0.94 | 5.90 | 0.01 | 0.98 | 0.90 |
| 20.09.2023 | 0.42 | 0.12 | 0.15 | 0.69 | 0.35 | 4.62 | 0.40 | 0.16 | 0.25 | 1.44 | 0.11 | 1.55 | 2.64 | 7.83 | 1.57 | 0.22 | 0.69 | 0.01 |
| 27.09.2023 | 0.48 | 0.12 | 0.21 | 0.75 | 0.26 | 5.30 | 0.40 | 0.16 | 0.49 | 1.36 | 0.07 | 1.43 | 1.37 | 2.01 | 8.18 | 0.00 | 0.84 | 0.18 |
| 04.10.2023 | 0.59 | 0.13 | 0.31 | 0.87 | 0.44 | 6.45 | 0.42 | 0.17 | 0.43 | 1.30 | 0.21 | 1.51 | 1.38 | 1.20 | 3.27 | 0.05 | 0.79 | 0.09 |
| 11.10.2023 | 0.48 | 0.09 | 0.28 | 0.68 | 0.45 | 5.27 | 0.30 | 0.09 | 0.34 | 0.95 | 0.13 | 1.08 | 0.78 | 0.00 | 3.07 | 0.06 | 0.89 | 0.39 |
| 18.10.2023 | 0.49 | 0.10 | 0.27 | 0.71 | 0.35 | 5.38 | 0.33 | 0.11 | 0.20 | 1.09 | 0.24 | 1.33 | 2.09 | 4.37 | 3.21 | 0.05 | 0.90 | 0.41 |
| 25.10.2023 | 0.35 | 0.05 | 0.25 | 0.46 | 0.32 | 3.88 | 0.16 | 0.02 | 0.19 | 0.53 | 0.15 | 0.68 | 0.83 | 0.40 | 4.52 | 0.02 | 0.98 | 0.91 |
| 08.11.2023 | 0.77 | 0.17 | 0.40 | 1.14 | 0.63 | 8.46 | 0.55 | 0.31 | 0.69 | 1.81 | 0.17 | 1.98 | 1.16 | 0.82 | 2.21 | 0.11 | 0.80 | 0.10 |
| 15.11.2023 | 0.25 | 0.02 | 0.19 | 0.30 | 0.21 | 2.74 | 0.08 | 0.01 | 0.10 | 0.24 | 0.16 | 0.40 | 0.78 | -0.62 | 17.59 | <.001 | 0.93 | 0.58 |
| 22.11.2023 | 0.58 | 0.17 | 0.20 | 0.97 | 0.38 | 6.42 | 0.58 | 0.33 | 0.44 | 1.98 | 0.10 | 2.08 | 2.03 | 4.43 | 3.88 | 0.03 | 0.90 | 0.41 |
| 29.11.2023 | 0.46 | 0.11 | 0.22 | 0.70 | 0.34 | 5.06 | 0.36 | 0.13 | 0.15 | 1.30 | 0.12 | 1.42 | 2.17 | 5.37 | 6.85 | 0.01 | 0.84 | 0.20 |
| 06.12.2023 | 0.31 | 0.03 | 0.24 | 0.38 | 0.29 | 3.40 | 0.10 | 0.01 | 0.08 | 0.40 | 0.17 | 0.57 | 1.65 | 4.19 | 17.71 | <.001 | 0.81 | 0.12 |
| 13.12.2023 | 0.55 | 0.05 | 0.43 | 0.66 | 0.54 | 6.00 | 0.18 | 0.03 | 0.20 | 0.55 | 0.20 | 0.75 | -0.84 | -0.06 | 5.85 | 0.01 | 0.80 | 0.11 |
| 20.12.2023 | 1.08 | 0.27 | 0.34 | 1.82 | 0.89 | 5.39 | 0.60 | 0.36 | 0.38 | 1.58 | 0.46 | 2.04 | 1.23 | 1.91 | 3.43 | 0.04 | 0.97 | 0.81 |

For the confidence interval mean. it was assumed that the sample means follow a t-distribution with N - 1 degrees of freedom;

LCL – lower confidence level; UCL – upper confidence level

*Note. Hₐ μ ≠ 0 for t-student and df=3; one-sample test

** W-test (Shapiro Wilk) a low p-value suggests a violation of the normality assumption

B)

| DATE | Mean  [g] | SE | LCL90% | UCL90% | Median | Sum [g] | SD | Variance | IQR | Range  [g] | Min.  [g] | Max. [g] | Skewness | Kurtosis | t-student* | p* | W** | p** |
| --- | --- | --- | --- | --- | --- | --- | --- | --- | --- | --- | --- | --- | --- | --- | --- | --- | --- | --- |
| 10.01.2024 | 1.08 | 0.27 | 0.34 | 1.82 | 0.89 | 5.39 | 0.60 | 0.36 | 0.38 | 1.58 | 0.46 | 2.04 | 1.23 | 1.91 | 3.43 | 0.04 | 0.97 | 0.81 |
| 17.01.2024 | 0.45 | 0.07 | 0.29 | 0.60 | 0.40 | 4.89 | 0.23 | 0.05 | 0.36 | 0.71 | 0.11 | 0.82 | 0.13 | -1.16 | 5.99 | 0.01 | 0.90 | 0.43 |
| 24.01.2024 | 0.45 | 0.06 | 0.32 | 0.57 | 0.49 | 4.92 | 0.19 | 0.04 | 0.28 | 0.53 | 0.14 | 0.67 | -0.66 | -1.03 | 3.35 | 0.04 | 0.86 | 0.27 |
| 30.01.2024 | 0.34 | 0.04 | 0.24 | 0.44 | 0.39 | 3.76 | 0.15 | 0.02 | 0.23 | 0.45 | 0.07 | 0.52 | -0.47 | -0.81 | 7.53 | 0.01 | 0.93 | 0.58 |
| 07.02.2024 | 0.41 | 0.05 | 0.30 | 0.53 | 0.34 | 4.53 | 0.17 | 0.03 | 0.22 | 0.56 | 0.22 | 0.78 | 1.03 | 0.82 | 3.36 | 0.04 | 0.78 | 0.07 |
| 14.02.2024 | 0.47 | 0.05 | 0.37 | 0.57 | 0.50 | 5.19 | 0.15 | 0.02 | 0.24 | 0.44 | 0.26 | 0.70 | -0.05 | -1.31 | 6.72 | 0.01 | 0.91 | 0.47 |
| 21.02.2024 | 0.34 | 0.05 | 0.24 | 0.45 | 0.30 | 3.78 | 0.15 | 0.02 | 0.18 | 0.49 | 0.12 | 0.61 | 0.26 | -0.53 | 5.11 | 0.02 | 0.87 | 0.30 |
| 28.02.2024 | 0.32 | 0.03 | 0.26 | 0.37 | 0.29 | 3.46 | 0.08 | 0.01 | 0.13 | 0.26 | 0.21 | 0.47 | 0.50 | -0.79 | 22.56 | <.001 | 0.83 | 0.16 |
| 06.03.2024 | 0.39 | 0.05 | 0.27 | 0.51 | 0.33 | 4.29 | 0.18 | 0.03 | 0.21 | 0.63 | 0.16 | 0.79 | 1.13 | 1.40 | 2.99 | 0.06 | 0.69 | 0.01 |
| 13.03.2024 | 0.18 | 0.06 | 0.05 | 0.30 | 0.15 | 1.92 | 0.19 | 0.04 | 0.14 | 0.68 | 0.03 | 0.71 | 2.48 | 7.09 | 1.60 | 0.21 | 0.77 | 0.05 |
| 20.03.2024 | 0.32 | 0.04 | 0.23 | 0.41 | 0.28 | 3.51 | 0.13 | 0.02 | 0.07 | 0.45 | 0.14 | 0.59 | 1.01 | 1.11 | 4.91 | 0.02 | 0.96 | 0.77 |
| 27.03.2024 | 0.38 | 0.03 | 0.31 | 0.45 | 0.39 | 4.14 | 0.10 | 0.01 | 0.13 | 0.34 | 0.14 | 0.48 | -1.26 | 1.62 | 18.87 | <.001 | 0.88 | 0.33 |
| 10.04.2024 | 0.62 | 0.19 | 0.19 | 1.04 | 0.41 | 6.78 | 0.63 | 0.40 | 0.18 | 2.21 | 0.27 | 2.48 | 3.11 | 9.96 | 2.05 | 0.13 | 0.73 | 0.02 |
| 17.04.2024 | 0.44 | 0.05 | 0.32 | 0.55 | 0.44 | 4.79 | 0.18 | 0.03 | 0.21 | 0.62 | 0.16 | 0.78 | 0.19 | 0.28 | 4.41 | 0.02 | 0.94 | 0.63 |
| 24.04.2024 | 0.81 | 0.21 | 0.34 | 1.29 | 0.62 | 8.92 | 0.71 | 0.50 | 0.47 | 2.16 | 0.17 | 2.33 | 1.58 | 1.47 | 2.49 | 0.09 | 0.79 | 0.08 |
| 30.04.2024 | 0.51 | 0.05 | 0.39 | 0.62 | 0.47 | 5.55 | 0.18 | 0.03 | 0.17 | 0.55 | 0.32 | 0.87 | 1.14 | 0.67 | 4.89 | 0.02 | 0.91 | 0.50 |
| 15.05.2024 | 0.55 | 0.16 | 0.19 | 0.90 | 0.45 | 6.01 | 0.53 | 0.28 | 0.17 | 1.88 | 0.22 | 2.10 | 3.06 | 9.80 | 1.73 | 0.18 | 0.73 | 0.02 |
| 18.05.2024 | 0.32 | 0.03 | 0.26 | 0.38 | 0.29 | 3.50 | 0.09 | 0.01 | 0.16 | 0.22 | 0.22 | 0.44 | 0.32 | -1.66 | 7.76 | 0.00 | 0.93 | 0.57 |
| 22.05.2024 | 0.43 | 0.05 | 0.31 | 0.54 | 0.45 | 4.67 | 0.18 | 0.03 | 0.25 | 0.56 | 0.18 | 0.74 | 0.14 | -0.69 | 3.58 | 0.04 | 0.91 | 0.49 |
| 29.05.2024 | 0.39 | 0.05 | 0.29 | 0.49 | 0.40 | 4.27 | 0.15 | 0.02 | 0.20 | 0.51 | 0.17 | 0.68 | 0.30 | -0.01 | 7.92 | 0.00 | 0.97 | 0.83 |
| 05.06.2024 | 0.49 | 0.06 | 0.35 | 0.62 | 0.48 | 5.34 | 0.20 | 0.04 | 0.16 | 0.72 | 0.21 | 0.93 | 1.04 | 1.77 | 7.30 | 0.01 | 0.98 | 0.90 |
| 12.06.2024 | 0.41 | 0.06 | 0.27 | 0.54 | 0.32 | 4.47 | 0.20 | 0.04 | 0.31 | 0.60 | 0.16 | 0.76 | 0.46 | -1.10 | 7.09 | 0.01 | 0.82 | 0.14 |
| 19.06.2024 | 0.72 | 0.11 | 0.48 | 0.96 | 0.59 | 7.89 | 0.35 | 0.13 | 0.52 | 0.99 | 0.31 | 1.30 | 0.56 | -1.08 | 6.24 | 0.01 | 0.86 | 0.24 |
| 26.06.2024 | 0.43 | 0.06 | 0.30 | 0.56 | 0.36 | 4.72 | 0.19 | 0.04 | 0.32 | 0.54 | 0.19 | 0.73 | 0.55 | -1.47 | 9.30 | 0.00 | 0.85 | 0.22 |
| 03.07.2024 | 0.41 | 0.07 | 0.25 | 0.56 | 0.36 | 4.50 | 0.23 | 0.05 | 0.27 | 0.71 | 0.18 | 0.89 | 1.07 | 0.47 | 8.09 | 0.00 | 0.75 | 0.04 |
| 10.07.2024 | 0.38 | 0.05 | 0.27 | 0.48 | 0.35 | 4.16 | 0.16 | 0.02 | 0.09 | 0.59 | 0.17 | 0.76 | 1.57 | 3.22 | 11.43 | 0.00 | 0.73 | 0.02 |
| 17.07.2024 | 0.33 | 0.04 | 0.23 | 0.42 | 0.34 | 3.57 | 0.13 | 0.02 | 0.21 | 0.38 | 0.11 | 0.49 | -0.38 | -1.14 | 3.93 | 0.03 | 0.99 | 0.96 |
| 24.07.2024 | 0.52 | 0.07 | 0.35 | 0.68 | 0.44 | 5.67 | 0.25 | 0.06 | 0.31 | 0.80 | 0.22 | 1.02 | 0.96 | 0.12 | 3.63 | 0.04 | 0.89 | 0.40 |
| 31.07.2024 | 0.49 | 0.10 | 0.27 | 0.71 | 0.39 | 5.42 | 0.32 | 0.11 | 0.12 | 1.18 | 0.27 | 1.45 | 3.06 | 9.78 | 7.86 | 0.00 | 0.97 | 0.87 |
| 07.08.2024 | 0.46 | 0.06 | 0.31 | 0.60 | 0.41 | 5.04 | 0.21 | 0.05 | 0.18 | 0.79 | 0.24 | 1.03 | 2.12 | 5.52 | 9.33 | 0.00 | 0.99 | 0.95 |
| 14.08.2024 | 0.52 | 0.11 | 0.28 | 0.76 | 0.36 | 5.73 | 0.35 | 0.12 | 0.33 | 1.13 | 0.14 | 1.27 | 1.25 | 0.81 | 2.15 | 0.12 | 0.73 | 0.02 |
| 21.08.2024 | 0.33 | 0.04 | 0.24 | 0.42 | 0.37 | 3.60 | 0.13 | 0.02 | 0.23 | 0.36 | 0.12 | 0.48 | -0.36 | -1.66 | 3.51 | 0.04 | 0.85 | 0.23 |
| 28.08.2024 | 0.75 | 0.13 | 0.47 | 1.02 | 0.63 | 8.20 | 0.41 | 0.17 | 0.44 | 1.34 | 0.30 | 1.64 | 1.20 | 0.76 | 2.69 | 0.07 | 0.87 | 0.29 |
| 04.09.2024 | 0.32 | 0.05 | 0.21 | 0.43 | 0.27 | 3.50 | 0.16 | 0.03 | 0.23 | 0.46 | 0.13 | 0.59 | 0.57 | -0.91 | 2.78 | 0.07 | 0.85 | 0.22 |
| 11.09.2024 | 0.56 | 0.07 | 0.40 | 0.73 | 0.46 | 6.19 | 0.24 | 0.06 | 0.35 | 0.79 | 0.19 | 0.98 | 0.40 | -0.82 | 5.41 | 0.01 | 0.77 | 0.06 |
| 02.10.2024 | 0.39 | 0.04 | 0.30 | 0.48 | 0.39 | 4.30 | 0.13 | 0.02 | 0.16 | 0.43 | 0.19 | 0.62 | 0.03 | -0.36 | 5.68 | 0.01 | 0.98 | 0.92 |
| 09.10.2024 | 0.45 | 0.07 | 0.30 | 0.59 | 0.41 | 4.91 | 0.22 | 0.05 | 0.30 | 0.65 | 0.14 | 0.79 | 0.18 | -1.02 | 4.02 | 0.03 | 0.93 | 0.59 |
| 16.10.2024 | 0.51 | 0.07 | 0.36 | 0.67 | 0.50 | 5.64 | 0.23 | 0.05 | 0.26 | 0.77 | 0.25 | 1.02 | 0.94 | 0.79 | 8.50 | 0.00 | 0.79 | 0.09 |
| 23.10.2024 | 0.44 | 0.06 | 0.31 | 0.57 | 0.45 | 4.39 | 0.18 | 0.03 | 0.25 | 0.54 | 0.13 | 0.67 | -0.47 | -0.86 | 3.28 | 0.05 | 0.94 | 0.68 |
| 30.10.2024 | 0.49 | 0.04 | 0.40 | 0.57 | 0.43 | 5.34 | 0.13 | 0.02 | 0.16 | 0.40 | 0.34 | 0.74 | 0.90 | -0.08 | 8.94 | 0.00 | 0.95 | 0.74 |
| 06.11.2024 | 0.49 | 0.07 | 0.34 | 0.64 | 0.43 | 5.38 | 0.22 | 0.05 | 0.17 | 0.82 | 0.23 | 1.05 | 1.61 | 3.71 | 6.44 | 0.01 | 0.94 | 0.68 |
| 13.11.2024 | 0.54 | 0.06 | 0.39 | 0.68 | 0.66 | 5.89 | 0.21 | 0.05 | 0.34 | 0.56 | 0.23 | 0.79 | -0.31 | -1.75 | 4.21 | 0.02 | 0.86 | 0.27 |
| 20.11.2024 | 0.52 | 0.08 | 0.35 | 0.69 | 0.51 | 5.75 | 0.25 | 0.06 | 0.34 | 0.75 | 0.21 | 0.96 | 0.54 | -0.79 | 3.46 | 0.04 | 0.90 | 0.44 |
| 27.11.2024 | 0.45 | 0.04 | 0.35 | 0.54 | 0.47 | 4.90 | 0.14 | 0.02 | 0.16 | 0.48 | 0.27 | 0.75 | 0.92 | 1.27 | 11.41 | 0.00 | 0.98 | 0.91 |
| 04.12.2024 | 0.48 | 0.06 | 0.36 | 0.61 | 0.41 | 5.31 | 0.19 | 0.03 | 0.26 | 0.55 | 0.29 | 0.84 | 0.83 | -0.40 | 16.61 | <.001 | 0.88 | 0.33 |
| 11.12.2024 | 0.49 | 0.04 | 0.39 | 0.59 | 0.45 | 5.39 | 0.14 | 0.02 | 0.09 | 0.52 | 0.35 | 0.87 | 2.15 | 5.33 | 17.08 | <.001 | 0.90 | 0.43 |
| 18.12.2024 | 0.43 | 0.05 | 0.32 | 0.54 | 0.41 | 4.75 | 0.17 | 0.03 | 0.16 | 0.57 | 0.23 | 0.80 | 1.06 | 1.33 | 6.94 | 0.01 | 0.95 | 0.72 |

For the confidence interval mean. it was assumed that the sample means follow a t-distribution with N - 1 degrees of freedom;

LCL – lower confidence level; UCL – upper confidence level

*Note. Hₐ μ ≠ 0 for t-student and df=3; one-sample test

** W-test (Shapiro Wilk) a low p-value suggests a violation of the normality assumption

IQR – interquartile range

**Supplementary Table 2.** Analysis of variance (one-way) for the weight of the pellet between single collection points.

| Groups of CPs in given date | | | Test statistic | p-value  for given test | Comparison among groups | Significance |
| --- | --- | --- | --- | --- | --- | --- |
| 5.07.23 | **>** | 12.07.23; 18.07.23; 13.03.24 | 2.31≤x≤2.56 | 0.012≥x≥0.021 | Fisher LSD and Dunnett’s tests | moderate to strong |
|  | **<** | 8.11.23; 20.12.23; 10.01.24; 24.04.24 | 2.27≤x≤3.75 | 0.02≥x≥0.0002 |  |  |
| 12.07.23 | **<** | 8.11.23; 20.12.23; 10.01.24*.* 24.04.24 19.06.24*.* 28.08.24 | 4.17≤x≤5.57 | 1.34E-5≥x≥3.80E-8 | Bonferoni and Fisher LSD tests | strong |
| 18.07.23 | **<** | 2.08.23; 8.11.23; 20.12.23; 10.01.24; 24.04.24; 19.06.24; 28.08.24 | 3.95≤x≤5.73 | 1.51E-5≥x≥1.63E-8 |  |  |

*** The highest weights in range 0.62 and 1.08g (2.08.23; 8.11.23; 20.12.23; 10.01.24; 10.04.24; 24.04.24; 28.08.24)
without significant differences between values

**Supplementary Table 3.** Matrices of positive A) and negative linear corelations B) for the weight of the pellet.

**A)**

|  | 30.06.23 | 12.07.23 | 2.08.23 | 20.09.23 | 27.09.23 | 15.11.23 | 7.02.24 | 29.05.24 | 5.06.24 | 10.07.24 |
| --- | --- | --- | --- | --- | --- | --- | --- | --- | --- | --- |
| 8.11.23 |  |  | 0.91 |  |  |  |  |  |  |  |
| 17.01.24 |  | 0.90 |  |  | 0.90 |  |  |  |  |  |
| 21.02.24 |  |  |  |  |  | 0.92 |  |  |  |  |
| 13.03.24 |  |  |  | 0.93 |  |  |  |  |  |  |
| 12.06.24 |  |  |  |  |  | 0.90 |  |  |  |  |
| 7.08.24 |  |  |  |  |  |  |  |  | 0.90 |  |
| 14.08.24 |  |  |  |  |  |  | 0.94 |  |  |  |
| 16.10.24 | **0.95** |  |  |  |  |  |  | 0.92 |  |  |
| 6.11.24 |  |  |  |  |  |  |  |  |  | **0.95** |

p < 0.001. one-side hypothesis and significances only for statistics values r>0.85

shaded cells – summer period

bolded – the highest positive correlations

**B)**

|  | 5.07.2023 | 2.08.2023 | 9.08.2023 | 18.10.2023 | 8.11.2023 |
| --- | --- | --- | --- | --- | --- |
| 21.02.2024 |  |  | -0.69 |  |  |
| 17.04.2024 |  |  | -0.71 |  |  |
| 30.04.2024 | -0.72 |  |  |  |  |
| 17.07.2024 |  | **-0.80** |  |  |  |
| 2.10.2024 |  |  |  |  | -0.72 |
| 13.11.2024 |  |  |  | -0.74 |  |

p < 0.01. one-side hypothesis and significances only for statistics values r>0.70

shaded cells – spring and summer period

bolded – the highest negative correlations

**Supplementary Table 4.** Frequency of presence for positive qPCRs in the pellet with simultaneous negative in the supernatant.

| Month | SCPs | EHEC | STEC | EPEC | EIEC | ETEC | *Shigella* type1 | vanA | VIM | met-oxa-R | MSSA | MRCoNS | Sum |
| --- | --- | --- | --- | --- | --- | --- | --- | --- | --- | --- | --- | --- | --- |
| May | AH | + | + |  | + |  | + |  | + |  |  |  | 5 |
|  | HCU |  |  |  |  |  |  | + |  |  |  |  | 1 |
| June | HCU |  |  | + |  |  |  |  |  | + |  |  | 2 |
|  | HE |  |  | + |  |  |  |  |  |  | + |  | 2 |
|  | WTP |  |  |  |  | + |  |  |  |  |  |  | 1 |
| June | HCU |  |  | + |  |  |  |  |  | + |  |  | 2 |
|  | HE |  |  | + |  |  |  |  |  |  | + |  | 2 |
|  | WTP |  |  |  |  | + |  |  |  |  |  |  | 1 |
| Aug | HCU |  |  | + |  |  |  |  |  | + |  |  | 2 |
|  | HE |  |  | + |  |  |  |  |  |  | + |  | 2 |
| Oct | AH |  |  |  |  |  |  |  |  |  | + |  | 1 |
|  | HCU |  |  |  |  |  |  |  |  |  | + |  | 1 |
|  | HE |  |  |  |  |  |  |  |  | + |  |  | 1 |
|  | WTP |  |  |  |  |  |  |  |  |  | + |  | 1 |
| Nov | HE |  |  |  |  |  |  |  |  | + |  |  | 1 |
| Dec | AH |  |  |  |  |  |  |  |  |  | + |  | 1 |
|  | HCU |  |  |  |  |  |  |  |  | + |  |  | 1 |
| **Jan** | AH |  |  | + |  |  |  |  |  |  | + |  | 2 |
|  | HCU |  |  |  |  |  |  |  |  |  | + |  | 1 |
|  | HE |  |  |  |  |  |  |  |  |  | + |  | 1 |
| **Feb** | AH |  |  | + |  |  |  |  |  |  |  |  | 1 |
|  | WTP |  |  |  |  |  |  |  |  |  |  | + | 1 |
| **Mar** | AH |  |  |  |  |  |  |  |  |  |  | + | 1 |
| **Apr** | AH |  |  |  |  |  |  |  |  |  |  | + | 1 |
|  | HCU |  |  |  | + |  |  |  |  | + |  |  | 2 |
| **May** | AH |  | + |  |  |  |  |  | + |  |  |  | 2 |
|  | HCU |  |  |  |  |  |  |  |  |  | + |  | 1 |
|  | HE |  |  |  |  |  |  | + |  | + | + |  | 3 |
|  | WTP |  |  |  |  |  |  |  |  | + |  | + | 2 |
| **Jul** | HCU |  |  |  |  |  |  |  |  | + |  |  | 1 |
|  | HE |  |  |  |  |  |  |  |  |  | + |  | 1 |
|  | WTP |  |  |  |  |  |  |  |  |  |  | + | 1 |
| **Aug** | HCU |  |  |  |  |  |  |  |  | + |  |  | 1 |
|  | HE |  |  |  |  |  |  |  |  |  | + |  | 1 |
|  | WTP |  |  |  |  |  |  |  |  |  |  | + | 1 |
| **Sept** | AH |  |  |  |  |  |  |  |  | + |  |  | 1 |
|  | HCU |  |  | + |  |  |  |  |  | + |  |  | 2 |
|  | WTP | + |  |  |  |  |  |  |  | + |  |  | 2 |
| **Oct** | AH |  |  | + |  | + |  |  |  |  | + |  | 3 |
|  | HCU |  |  |  |  | + |  |  |  |  |  |  | 1 |
|  | HE |  |  | + |  |  |  |  |  |  |  |  | 1 |
|  | WTP |  |  | + |  |  |  |  |  |  |  |  | 1 |
| **Nov** | AH |  |  | + |  |  |  |  |  |  |  |  | 1 |
|  | HCU |  |  |  |  |  |  |  |  |  |  | + | 1 |
|  | HE |  |  | + |  |  |  |  |  |  |  |  | 1 |
|  | WTP | + |  | + | + |  | + |  |  |  |  | + | 5 |
| Sum |  | 3 | 2 | 15 | 3 | 4 | 2 | 2 | 2 | 13 | 15 | 8 |  |

Shaded cells with + indicates positive qPCR reaction in the pellet and negative in the supernatant in one sample.

Regular and **Bolded** text indicate investigation during 2023 and 2024, respectively.
